# Polymorphisms in the BER and NER pathways and their influence on survival and toxicity in never-smokers with lung cancer

**DOI:** 10.1038/s41598-020-78051-5

**Published:** 2020-12-03

**Authors:** Ana Casal-Mouriño, Alberto Ruano-Ravina, María Torres-Durán, Isaura Parente-Lamelas, Mariano Provencio-Pulla, Olalla Castro-Añón, Iria Vidal-García, José Abal-Arca, María Piñeiro-Lamas, Alberto Fernández-Villar, Luis Valdés-Cuadrado, Juan Miguel Barros-Dios, Mónica Pérez-Ríos

**Affiliations:** 1 Department of Pneumology, Santiago de Compostela University Clinical Teaching Hospital, Santiago de Compostela, Spain; 2 Department of Preventive Medicine and Public Health, University of Santiago de Compostela, C/San Francisco s/n, 15782 Santiago de Compostela, Spain; 3 Consortium for Biomedical Research in Epidemiology and Public Health (CIBER en Epidemiología and Salud Pública-CIBERESP), Madrid, Spain; 4 Department of Pneumology, Vigo University Teaching Hospital Complex, Vigo, Spain; 5 Department of Pneumology, Ourense University Teaching Hospital Complex, Ourense, Spain; 6 Department of Oncology, Puerta de Hierro University Teaching Hospital, Madrid, Spain; 7 Department of Pneumology, Lucus Augusti Hospital, Lugo, Spain; 8 Department of Pneumology, A Coruña University Teaching Hospital Complex, Vigo, Spain; 9 Interdisciplinary Neumology Research Group, Health Research Institute of Santiago de Compostela (Instituto Investigación Sanitaria de Santiago de Compostela/IDIS), Santiago de Compostela, Spain; 10 C013 Group-Health Research Institute of Santiago de Compostela (Instituto Investigación Sanitaria de Santiago de Compostela/IDIS), Santiago de Compostela, Spain

**Keywords:** Diseases, Oncology

## Abstract

Polymorphisms in DNA repair pathways may play a relevant role in lung cancer survival in never-smokers. Furthermore, they could be implicated in the response to chemotherapy and toxicity of platinum agents. The aim of this study was to evaluate the influence of various genetic polymorphisms in the BER and NER DNA repair pathways on survival and toxicity in never-smoker LC patients. The study included never-smokers LC cases diagnosed from 2011 through 2019, belonging to the Lung Cancer Research In Never Smokers study. A total of 356 never-smokers cases participated (79% women; 83% adenocarcinoma and 65% stage IV). Survival at 3 and 5 years from diagnosis was not associated with genetic polymorphisms, except in the subgroup of patients who received radiotherapy or chemo-radiotherapy, and presented with ERCC1 rs3212986 polymorphism. There was greater toxicity in those presenting OGG1 rs1052133 (CG) and ERCC1 rs11615 polymorphisms among patients treated with radiotherapy or chemo-radiotherapy, respectively. In general, polymorphisms in the BER and NER pathways do not seem to play a relevant role in survival and response to treatment among never-smoker LC patients.

## Introduction

Lung cancer (LC) is an important public health problem, ranking as the leading cause of cancer-related death and causing approximately 1.4 million deaths annually worldwide^1^. Although smoking habit is the principal risk factor for LC, up to 15% of all LC cases in men and 53% in women occur among never-smokers, with lung cancer in never-smokers being regarded as a different clinical entity in which residential radon is the principal etiologic agent^2–4^.

LC survival has hardly improved in recent years, standing at around 10–25% at 5 years from diagnosis^5^. Specific survival of never-smoker patients is not accurately known but ranges from 7 through 45 months^6^. On the whole, little is known about how genetic susceptibility may influence lung cancer survival. This ignorance is greater when it comes to never-smokers, and is greater still when genetic susceptibility is combined with the treatments received.

Genetic variability may play a fundamental role in LC survival among individuals who receive a similar treatment. Some genetic polymorphisms have been linked to the response to certain chemotherapy treatments, something that might partly account for interindividual variability in the response to a given drug^8–10^. To date, however, there are few studies which have specifically analyzed the role of these polymorphisms in survival, and even fewer in never-smokers.

There are two DNA repair mechanisms, known as base excision repair (BER) and nucleotide excision repair (NER), respectively. The NER mechanism is the process that eliminates adducts of damaged DNA, and forms a major part of cellular defense mechanism against neoplasms, mutagenesis and cytotoxicity. The BER mechanism, on the other hand, is tasked with maintaining genome integrity, correcting the modifications in the DNA bases, such as those that produce alkylating chemotherapeutic agents. The two mechanisms differ in that NER is capable of repairing DNA fragments with a length of 30 bases, whereas BER repairs fragments having a length of 1 to 6 bases^11,12^. The polymorphisms in genes involved in these molecular pathways can contribute to variability in lung cancer survival^13^. In particular, platinum compounds are DNA alkylating agents, which means that, theoretically speaking, subjects with better DNA repair might respond worse to platinum agents, since they would better repair the DNA in the cancer cell in the face of aggression from these drugs^14^. There are studies which show that polymorphisms in genes involved in DNA repair (like ERCC1: excision repair 1 gene) may influence the non-small cell lung cancer prognosis^15^.

Alpha1-antitrypsin (AAT) is another acute phase protein and an archetype member of serpin superfamily, well recognized for its role as a serine protease inhibitor, but also known as an inhibitor of caspase-3, anti-apoptotic and immunomodulatory protein. The salvage gene of AAT is designated PiM (proteinase inhibitor M) and the two most frequent deficient alleles are PiS, which expresses approximately 50% to 60% of AAT, and PiZ, which expresses approximately 10% to 20% of AAT. In clinical practice, most (95%) of AAT deficiency-related diseases are linked with PiZZ phenotype. It is known that AAT plays a significant role in LC tumorigenesis in never smokers^16^. Elevated levels of plasma alpha1-antitrypsin (AAT) correlate with a poor lung cancer prognosis^17^.

In addition, glutation-S-transferases (GSTs) are a group of phase II detoxification enzymes implicated in cellular defense against external agents. Within the GST superfamily, the GSTT1 and GSTM1 genes are deleted in approximately 20% and 50% of the Caucasian population, respectively^18^. Depending on patients’ GST activity, their response to radiotherapy or chemotherapy, particularly in the case of platinum derivatives, might be different^19^. Due to this same mechanism, the deletion of these genes will have an influence on the side effects of the treatment administered^20,21^. It is postulated that these polymorphisms may influence survival, though the evidence of this effect is more limited**.**

A number of these polymorphisms have been associated with the metabolization of platinum-based chemotherapeutic compounds, and -by extension- with the efficacy that this type of treatment may have on LC patients’ survival. A recent meta-analysis reports that in patients with non-small cell lung cancer (NSCLC), the ERCC1 (rs11615), XRCC1 (X-ray repair cross complementing 1: rs25487, rs1799782) and XPD (xeroderma pigmentosum group D: rs13181) polymorphisms are predictive factors of effectiveness in response to chemotherapy based on platinum regimens^22^.

Accordingly, the aim of this study was to evaluate the influence of genetic polymorphisms (GSTM1, GSTT1, XRCC1 –rs25487-, ERCC1 –rs11615, rs3212986, ERCC2 –rs13181-, XRCC3 –rs861539-, OGG1 –rs1052133- and Alpha-1-antitrypsin (AAT) mainly located in the BER and NER DNA repair pathways on survival and toxicity among never-smoker LC patients.

## Methods

### Study-design and setting

A multicenter, hospital-based, case–control study performed in never-smokers, (Lung Cancer Research In Never Smokers—LCRINS) was conducted at 7 hospitals in Galicia, northwest Spain^23^. Patient recruitment took place from January 2011 through July 2019, and only included cases with histologic confirmation of LC who were never-smokers according to the WHO definition, i.e., anyone who has smoked: (1) fewer than 100 cigarettes in his/her lifetime; or (2) less than 1 cigarette per day during a period of no less than 6 months. All cases were over 18 years, without any upper age limit. Subjects with small cell lung cancer were excluded from this study, since the disease’s characteristics are different to those of other types of LC. The study protocol was approved by the Ethics Committee at the Santiago de Compostela Healthcare area (reference 2010/295), and informed consent was obtained from all patients. All methods were performed in accordance with the relevant guidelines and regulations.

### Data-collection

All patients were interviewed by trained researchers, who administered a questionnaire used in a number of previous studies ^23,24^ to collect data on LC risk factors. Exposure to environmental tobacco smoke was assessed through personal interview and included all persons living with the participant in the last 20 years. Exposure to residential radon was also measured with alpha-track detectors. The detector readings were processed under standard conditions at the Galician Radon Laboratory (School of Medicine, Santiago de Compostela, Spain), a facility officially certified by the National Accreditation Entity of Spain (https://www.radon.gal).

### Patients follow-up and evaluation of treatments and survival

All patients were followed up until date of death or last medical visit. Follow-up was conducted by consulting the electronic medical records, reviewed until July 2019. Follow-up time was calculated as the period of time between date of diagnosis and date of death or last visit. For all patients, we obtained histologic type, stage at diagnosis, and treatments received (chemotherapy, radiotherapy, chemo/radiotherapy, surgery). Special emphasis was laid on the type of chemotherapy treatment received. Data were also obtained on toxicity (overall and by type). Validated toxicity evaluation scores were used, such as that designed by the National Cancer Institute of Canada and the Eastern Cooperative Oncology Group.

### Laboratory methods

A 3-ml blood sample was obtained from all participants, and used for determination of different genetic polymorphisms. Samples were stored at – 84 ºC until time of analysis, which was performed by the National Genotyping Center (Centro Nacional de Genotipado-CeGen) at the University of Santiago de Compostela, Spain. The genetic analyses determined the complete deletion of the GSTM1 and GSTT1 genes, XRCC1 –rs25487-, ERCC1 –rs11615, rs3212986-, ERCC2 –rs13181-, XRCC3 –rs861539-, OGG1 –rs1052133- and AAT deficiency. The technology used was Agena Bioscience’s MassARRAY system, which enables determination of specific DNA variants, single nucleotide polymorphisms (SNPs), as well as small insertions and deletions. Polymerase chain reaction (PCR) was followed by iPLEX extension reactions. Reactions took place in 384 separate wells, and the extension products were transferred to a SpectroCHIP Array and analyzed with MALDI-TOF mass spectrometry.

### Statistical analysis

Firstly, a univariate analysis was performed, and survival of never-smoker patients was analyzed using Cox Regression in which the dependent variable was survival at 3 and 5 years of diagnosis taking into account the different polymorphisms analyzed. A multivariate Cox regression was further performed adjusted for age, sex, passive exposure to tobacco smoke, residential radon, and stage at diagnosis. The reference group for analyzing the data on the different genotypes was homozygosity for the most common allele (wild-type). In addition, we calculated survival according to the presence of genetic polymorphisms (survival, both overall and specific of patients in stage IV at diagnosis), as well as the effect of polymorphisms on toxicity to chemotherapy or other treatments. p-value was considered significant when it turned below 0.05. Analysis of each specific type of toxicity was performed with the aid of the Chi-square test, due to the low frequency of some types of toxicity and the polymorphisms analyzed. As multiple comparisons have been done, false discovery rate (FDR) was calculated in order to consider statistical power. We obtained survival graphs for some polymorphisms at 3 and 5-years from diagnosis. All analyses were performed using the IBM SPSS v22 software program (IBM, Armonk, NY, USA; https://www.ibm.com/analytics/spss-statistics-software).

## Results

The study included 356 never-smoker cases, 79% of whom were women. Mean age at diagnosis was similar in both sexes, the predominant histologic type was adenocarcinoma (83%), and the most frequent stage at diagnosis was IV (65%). 25% of all patients received chemotherapy, 11% received radiotherapy, and 28% received chemo-radiotherapy. The remaining patients received either palliative treatment or treatments directed at specific molecular targets. Table 1 shows the characteristics of the participants along with the distribution of the polymorphisms analyzed.

**Table 1:** Description of the sample.

Variable	Frequency (%)
**Sex**	
Men	76 (21.3)
Women	280 (78.7)
**Age at diagnosis (mean; median (P**_**25**_**–P**_**75**_**))**	
Overall	68; 70 (60–78)
Men	67.4; 67.5 (59.8–79.3)
Women	68.2; 71 (60–78)
Residential radon concentration (mean; median (P_25_–P_75_))	256.5; 178 (109–330.5)
**Histologic type**	
Adenocarcinoma	295 (82.9)
Squamous	34 (9.6)
Large cell	8 (2.2)
Other	19 (5.3)
**Diagnostic stage**	
I	42 (12.1)
II	23 (6.6)
III	58 (16.7)
IV	225 (64.6)
**Environmental tobacco smoke exposure**	153 (43.3)
**Mutation data**	
EGFR	130 (35.2)
ALK	21 (5.7)
ROS1	1 (0.3)
**Treatments**	
Chemotherapy	85 (24.7)
Radiotherapy	37 (10.8)
Chemo-radiotherapy	94 (27.3)
Others	128 (37.2)
**GSTM1**	
Salvage gene/heterozygous	86 (48.3)
Homozygous	92 (51.7)
**GSTT1**	
Salvage gene/heterozygous	128 (74.4)
Homozygous	44 (25.6)
**XRCC1 rs25487**	
GG	155 (47)
AG	132 (40)
AA	43 (13)
**ERCC1 rs11615**	
CC	55 (16.7)
CT	163 (49.4)
TT	112 (33.9)
**ERCC1 rs3212986**	
GG	177 (53.6)
GT	131 (39.7)
TT	22 (6.7)
**ERCC2 rs13181**	
TT	140 (42.4)
GT	154 (46.7)
GG	36 (10.9)
**XRCC3 rs861539**	
CC	120 (36.4)
TC	168 (50.9)
TT	42 (12.7)
**OGG1 rs1052133**	
CC	204 (61.8)
CG	109 (33)
GG	17 (5.2)
**Alpha-1-antytripsin**	
MM	235 (71.6)
MS	72 (22)
MZ	11 (3.4)
SZ	3 (0.9)
SS	7 (2.1)
**Type of toxicity**^a^	
Anti-EGFR toxicoderma	48 (13)
Neutropenia	19 (5.1)
Hepatotoxicity	3 (0.8)
Diarrhea	9 (2.4)
Nausea/vomiting	20 (5.4)

aTypes of toxicity with a frequency higher than 2%. Hepatotoxicity was present in 0.8% of cases.

Survival at 5 years from diagnosis according to the treatment received, along with the effect of each of the polymorphisms analyzed, is shown in Table 2 (data for survival at 3 years from diagnosis is shown in supplemental Table S1). In the group of patients who received chemotherapy, no association was observed between any of the polymorphisms analyzed and 5-year survival. In the group of patients who received other types of treatment, there was an association between survival at 5 years and presence of the ERCC1 rs3212986 (TT) polymorphism. Supplemental Fig. 1 shows 5-year lung cancer survival according to the treatment received and polymorphisms analyzed.

**Table 2:** Survival at 5 years of diagnosis, according to treatment received and type of polymorphism.

Variable	Chemotherapy (179 patients and 136 events)				Other treatments (112 patients and 69 events)			
	Crude HR (95% CI)	p-value	Adjusted HR^a^ (95% CI)	p-value	Crude HR (95% CI)	p-value	Adjusted HR^a^ (95% CI)	p-value
**GSTM1**								
Salvage gene/heterozygous	1 (–)		1 (–)		1 (–)		1 (–)	
Homozygous	1.12 (0.71–1.76)	0.63	1.22 (0.77–1.94)	0.39	0.56 (0.29–1-07)	0.08	0.83 (0.36–1.88)	0.65
**GSTT1**								
Salvage gene/heterozygous	1 (–)		1 (–)		1 (–)		1 (–)	
Homozygous	1.34 (0.81–2.21)	0.26	1.35 (0.81–2.26)	0.25	0.9 (0.41–1.98)	0.79	1.17 (0.48–2.84)	0.72
**XRCC1 rs25487**								
GG	1 (–)		1 (–)		1 (–)		1 (–)	
AG	1.2 (0.81–1.79)	0.36	1.2 (0.8–1.79)	0.37	1.09 (0.63–1.87)	0.84	1.54 (0.84–2.82)	0.21
AA	1.21 (0.7–2.1)	0.48	1.58 (0.89–2.79)	0.12	0.82 (0.29–2.32)	0.66	1.12 (0.32–3.86)	0.91
**ERCC1 rs11615**								
CC	1 (–)		1 (–)		1 (–)		1 (–)	
CT	0.89 (0.54–1.46)	0.64	1.06 (0.64–1.74)	0.83	0.58 (0.29–1.19)	0.14	0.76 (0.35–1.65)	0.52
TT	0.62 (0.36–1.06)	** < 0.001**	0.8 (0.46–1.4)	0.44	0.7 (0.34–1.45)	0.41	0.97 (0.45–2.09)	0.94
**ERCC1 rs3212986**								
GG	1 (–)		1 (–)		1 (–)		1 (–)	
GT	1.29 (0.88–1.89)	0.19	1.08 (0.73–1.61)	0.69	1.19 (0.68–2.07)	0.63	1.02 (0.57–1.82)	0.95
TT	1.75 (0.89–3.45)	0.11	1.64 (0.83–3.25)	0.16	3.65 (1.56–8.51)	** < 0.001**	3.28 (1.33–8.11)	**0.01**
**ERCC2 rs13181**								
TT	1 (–)		1 (–)		1 (–)		1 (–)	
GT	0.83 (0.56–1.23)	0.36	0.93 (0.62–1.38)	0.71	0.75 (0.42–1.32)	0.39	1.05 (0.56–1.96)	0.74
GG	1.22 (0.68–2.19)	0.51	1.12 (0.62–2.03)	0.71	1.56 (0.74–3.33)	0.25	1.94 (0.86–4.4)	0.11
**XRCC3 rs861539**								
CC	1 (–)		1 (–)		1 (–)		1 (–)	
TC	0.9 (0.6–1.35)	0.61	1 (0.67–1.5)	0.99	0.84 (0.49–1.45)	0.62	1 (0.56–1.79)	0.91
TT	0.65 (0.36–1.16)	0.15	0.88 (0.49–1.58)	0.66	0.93 (0.38–2.27)	0.87	0.99 (0.4–2.44)	0.96
**OGG1 rs1052133**								
CC	1 (–)		1 (–)		1 (–)		1 (–)	
CG	1.05 (0.71–1.55)	0.81	1 (0.68–1.48)	0.99	0.83 (0.47–1.47)	0.47	0.56 (0.3–1.03)	**0.05**
GG	2.28 (1.04–4.97)	**0.04**	1.63 (0.73–3.62)	0.23	3.49 (1.05–11.54)	**0.05**	1.32 (0.39–4.42)	0.70
**AAT**								
MM	1 (–)		1 (–)		1 (–)		1 (–)	
MS	1.19 (0.78–1.8)	0.42	1.03 (0.66–1.6)	0.91	1.42 (0.77–2.61)	0.30	0.93 (0.48–1.8)	0.73
MZ	0.65 (0.09–4.7)	0.67	0.98 (0.13–7.34)	0.98	1.36 (0.33–5.62)	0.70	1.19 (0.27–5.33)	0.82
SZ	5.65 (0.76–41.81)	0.09	3.3 (0.43–25.09)	0.25	2 (0.27–14.69)	0.51	2 (0.24–16.49)	0.50
SS	0.37 (0.05–2.69)	0.33	0.45 (0.06–3.29)	0.43	0.77 (0.11–5.58)	0.78	3.52 (0.42–29.55)	0.25

aAdjusted for age, sex, environmental tobacco smoke, indoor radon exposure, and stage at diagnosis.

Table 3 shows survival at 5 years from diagnosis for patients in stage IV according to the treatment received and polymorphisms analyzed (data for survival at 3 years from diagnosis is shown in supplemental: Table S2). In the group of stage IV patients who received chemotherapy, an association between survival at 5 years and presence of the AAT (MZ) polymorphism can be seen, while for the ERCC1 rs3212986 polymorphism the association was close to statistical significance.

**Table 3:** Survival at 5 years, according to the different polymorphisms and treatment received for patients in Stage IV.

Variable	Chemotherapy (119 patients and 106 events)				Other treatments (58 patients and 49 events)			
	Crude HR (95% CI)	p-value	Adjusted HR^a^ (95% CI)	p-value	Crude HR (95% CI)	p-value	Adjusted HR^a^ (95% CI)	**p-value**
**GSTM1**								
Salvage gene/heterozygous	1 (–)		1 (–)		1 (–)		1 (–)	
Homozygous	1.34 (0.8–2.24)	0.26	1.4 (0.83–2.33)	0.20	1.01 (0.45–2.28)	0.97	1.11 (0.43–2.87)	0.82
**GSTT1**								
Salvage gene/heterozygous	1 (–)		1 (–)		1 (–)		1 (–)	
Homozygous	1.31 (0.73–2.33)	0.36	1.32 (0.74–2.35)	0.35	1.16 (0.48–2.8)	0.75	1.45 (0.53–3.95)	0.46
**XRCC1 rs25487**								
GG	1 (–)		1 (–)		1 (–)		1 (–)	
AG	1.19 (0.76–1.86)	0.44	1.18 (0.76–1.84)	0.46	1.22 (0.63–2.37)	0.56	1.46 (0.69–3.09)	0.32
AA	1.84 (0.96–3.55)	0.07	1.73 (0.88–3.37)	0.11	3.19 (1.06–9.64)	**0.04**	2.28 (0.61–8.57)	0.22
**ERCC1 rs11615**								
CC	1 (–)		1 (–)		1 (–)		1 (–)	
CT	0.97 (0.57–1.66)	0.92	1 (0.59–1.7)	0.99	0.74 (0.32–1.72)	0.49	0.66 (0.27–1.63)	0.37
TT	0.73 (0.4–1.34)	0.31	0.75 (0.41–1.38)	0.36	1.11 (0.49–2.51)	0.80	1.1 (0.47–2.53)	0.83
**ERCC1 rs3212986**								
GG	1 (–)		1 (–)		1 (–)		1 (–)	
GT	1.19 (0.77–1.82)	0.43	1.14 (0.73–1.76)	0.57	1.11 (0.56–2.17)	0.77	0.94 (0.47–1.86)	0.85
TT	1.56 (0.69–3.52)	0.28	1.53 (0.68–3.45)	0.30	1.87 (0.62–5.59)	0.26	2.86 (0.9–9.12)	0.08
**ERCC2 rs13181**								
TT	1 (–)		1 (–)		1 (–)		1 (–)	
GT	0.85 (0.54–1.34)	0.49	0.85 (0.54–1.33)	0.47	0.78 (0.39–1.58)	0.49	1.01 (0.49–2.08)	0.97
GG	1.06 (0.56–2.01)	0.85	1.05 (0.55–1.98)	0.89	1.08 (0.45–2.58)	0.86	1.62 (0.64–4.06)	0.30
**XRCC3 rs861539**								
CC	1 (–)		1 (–)		1 (–)		1 (–)	
TC	0.97 (0.63–1.51)	0.90	0.98 (0.63–1.52)	0.93	1.11 (0.58–2.13)	0.75	1.2 (0.62–2.33)	0.59
TT	0.77 (0.37–1.6)	0.48	0.78 (0.37–1.64)	0.51	0.97 (0.28–3.34)	0.96	0.76 (0.22–2.69)	0.67
**OGG1 rs1052133**								
CC	1 (–)		1 (–)		1 (–)		1 (–)	
CG	1.09 (0.7–1.68)	0.71	1.07 (0.69–1.66)	0.75	0.77 (0.38–1.57)	0.47	0.56 (0.27–1.17)	0.12
GG	1.83 (0.78–4.28)	0.16	1.66 (0.69–3.97)	0.25	1.73 (0.52–5.83)	0.37	1.28 (0.38–4.34)	0.69
**AAT**								
MM	1 (–)		1 (–)		1 (–)		1 (–)	
MS	1.12 (0.7–1.79)	0.6507	1.07 (0.65–1.76)	0.7832	1.36 (0.64–2.89)	0.4172	1 (0.46–2.18)	0.99
MZ	7.75 (1.01–59.67)	0.0494	6.89 (0.86–55.45)	0.0695	0.51 (0.07–3.79)	0.5112	0.83 (0.1–6.6)	0.87
SZ	4.64 (0.62–34.7)	0.1351	4.15 (0.53–32.31)	0.174	0.93 (0.13–6.86)	0.9407	1.28 (0.16–10.39)	0.82
SS	0.57 (0.08–4.16)	0.583	0.61 (0.08–4.49)	0.628	–		–	

^a^ Adjusted for age, sex, environmental tobacco smoke, and indoor radon exposure.

Table 4 shows toxicity data according to the treatment received for each of the polymorphisms analyzed. No association was observed with the polymorphisms and toxicity due to chemotherapy, or for the group of patients who received platinum-based chemotherapy. A significant association was observed for patients who received radiotherapy and presented with the OGG1 rs1052133 (CG) polymorphism, and for those who received chemo-radiotherapy and presented with the ERCC1 rs11615 polymorphism (data shown in supplemental: Table S3). The most frequent toxicities were toxicoderma due to anti-epidermal growth factor receptor (EGFR) treatment, and the presence of nausea and vomiting (Supplemental Table S4). The presence of nausea and vomiting was statistically significantly associated with absence of the GSTT1 gene. As multiple comparisons have been done, false discovery rate (FDR) was calculated in order to consider statistical power (Supplemental Fig. 2).

**Table 4:** Toxicity for the different polymorphisms, according to treatment received (chemotherapy or chemotherapy with platinum).

Variable	Chemotherapy toxicity						Chemotherapy with platinum toxicity					
	Cases, n (%)	Controls, n (%)	Crude OR (95% CI)	p-value	Adjusted OR^a^ (95% CI)	p-value	Cases, n (%)	Controls, n(%)	Crude OR (95% CI)	p-value	Adjusted OR^a^ (95% CI)	p-value
**GSTM1**												
Salvage gene/heterozygous	10 (50)	11 (40.7)	1 (–)		1 (–)		8 (47.1)	9 (39.1)	1 (–)		1 (–)	
Homozygous	10 (50)	16 (59.3)	0.69 (0.21–2.21)	0.53	0.51 (0.12–2.19)	0.37	9 (52.9)	14 (60.9)	0.72 (0.2–2.59)	0.62	0.46 (0.08–2.41)	0.36
**GSTT1**												
Salvage gene/heterozygous	12 (60)	22 (78.6)	1 (–)		1 (–)		11 (64.7)	19 (79.2)	1 (–)		1 (–)	
Homozygous	8 (40)	6 (21.4)	2.44 (0.69–9.09)	0.17	5.19 (0.9–47.52)	0.09	6 (35.3)	5 (20.8)	2.07 (0.51–8.8)	0.31	3.37 (0.48–36.41)	0.25
**XRCC1 rs25487**												
GG	16 (51.6)	16 (34.8)	1 (–)		1 (–)		15 (55.6)	14 (35.9)	1 (–)		1 (–)	
AG	12 (38.7)	20 (43.5)	0.6 (0.22–1.62)	0.31	0.62 (0.21–1.84)	0.39	10 (37)	18 (46.2)	0.75 (0.44–1.29)	0.23	0.65 (0.35–1.18)	0.23
AA	3 (9.7)	10 (21.7)	0.3 (0.06–1.19)	0.11	0.67 (0.12–3.31)	0.63	2 (7.4)	7 (17.9)	0.56 (0.23–1.28)	0.13	0.52 (0.19–1.32)	0.54
**ERCC1 rs11615**												
CC	6 (19.4)	8 (17.4)	1 (–)		1 (–)		5 (18.5)	7 (17.9)	1 (–)		1 (–)	
CT	13 (41.9)	24 (52.2)	0.72 (0.21–2.61)	0.61	0.89 (0.2–4.01)	0.88	10 (37)	21 (53.8)	1.2 (0.58–2.64)	0.56	1.27 (0.55–3.11)	0.89
TT	12 (38.7)	14 (30.4)	1.14 (0.31–4.37)	0.84	1.1 (0.22–5.55)	0.91	12 (44.4)	11 (28.2)	2.07 (0.96–4.67)	0.56	2.02 (0.84–5.13)	0.55
**ERCC1 rs3212986**												
GG	16 (51.6)	26 (56.5)	1 (–)		1 (–)		16 (59.3)	22 (56.4)	1 (–)		1 (–)	
GT	14 (45.2)	17 (37)	1.34 (0.52–3.46)	0.54	1.24 (0.43–3.57)	0.68	10 (37)	15 (38.5)	0.78 (0.46–1.33)	0.87	0.82 (0.45–1.49)	0.76
TT	1 (3.2)	3 (6.5)	0.54 (0.03–4.65)	0.61	1.76 (0.06–52.31)	0.71	1 (3.7)	2 (5.1)	1.1 (0.38–2.97)	0.77	1.47 (0.43–4.82)	0.76
**ERCC2 rs13181**												
TT	14 (45.2)	22 (47.8)	1 (–)		1 (–)		12 (44.4)	20 (51.3)	1 (–)		1 (–)	
GT	17 (54.8)	19 (41.3)	0.71 (0.28–1.81)	0.48	1.57 (0.54–4.77)	0.41	15 (55.6)	14 (35.9)	1.12 (0.66–1.92)	0.27	0.78 (0.42–1.43)	0.33
GG	–	5 (10.9)	–		–		–	5 (12.8)	–		–	
**XRCC3 rs861539**												
CC	12 (38.7)	21 (45.7)	1 (–)		1 (–)		10 (37)	18 (46.2)	1 (–)		1 (–)	
TC	17 (54.8)	16 (34.8)	1.86 (0.7–5.07)	0.22	2.38 (0.78–7.7)	0.13	15 (55.6)	14 (35.9)	1.32 (0.75–2.32)	0.23	1.3 (0.7–2.44)	0.20
TT	2 (6.5)	9 (19.6)	0.39 (0.05–1.83)	0.27	0.15 (0.01–1.13)	0.11	2 (7.4)	7 (17.9)	1.59 (0.68–3.64)	0.46	1.86 (0.71–4.82)	0.20
**OGG1 rs1052133**												
CC	19 (61.3)	29 (63)	1 (–)		1 (–)		16 (59.3)	24 (61.5)	1 (–)		1 (–)	
CG	11 (35.5)	15 (32.6)	1.12 (0.42–2.95)	0.82	1.58 (0.53–4.86)	0.41	11 (40.7)	14 (35.9)	1.12 (0.65–1.93)	0.75	1.35 (0.74–2.47)	0.31
GG	1 (3.2)	2 (4.3)	0.76 (0.03–8.51)	0.83	0.96 (0.04–13.15)	0.98	–	1 (2.6)	–		–	
**Alpha-1-antytripsin**												
MM	25 (83.3)	27 (58.7)	1 (–)		1 (–)		22 (84.6)	24 (61.5)	1 (–)		1 (–)	
MS	4 (13.3)	16 (34.8)	0.27 (0.07–0.85)	0.04	0.15 (0.02–0.66)	0.02	3 (11.5)	12 (30.8)	0.73 (0.37–1.37)	0.07	0.61 (0.28–1.26)	0.08
MZ	1 (3.3)	–	–		–		1 (3.8)	–	–		–	
SZ	–	1 (2.2)	–		–		–	1 (2.6)	–		–	
SS	–	2 (4.3)	–		–		–	2 (5.1)	–		–	

^a^ Adjusted for age, sex, environmental tobacco smoke, and indoor radon exposure.

## Discussion

LC survival at 5 years from diagnosis in never-smokers does not seem to be clearly influenced by polymorphisms in genes implicated in DNA repair mediated by the BER or NER pathways, or by deletion of GSTM1 and GSTT1 genes. This apparent absence of effect is maintained for subjects treated with platinum compounds. On the other hand, the ERCC1 rs3212986 polymorphism does show greater toxicity for patients who have received radiotherapy or combined chemo-radiotherapy. Similarly, patients who received these treatments (radiotherapy or combined chemo-radiotherapy) presented with greater toxicity in the presence of the OGG1 rs1052133 (CG) and ERCC1 rs11615 polymorphisms, respectively. There was greater digestive toxicity (nausea and vomiting) in patients with GSTT1 deleted. In terms of sample size, it should be noted that the present study has one of the greatest sample sizes of never-smoker cases in whom these polymorphisms have been analyzed. Moreover, it is the only study to adjust its results for residential radon exposure.

DNA damage repair mechanisms in cancer are of various types, i.e., NER and BER^25^. NER-type repair begins with examination of the area of DNA damaged, and the genes involved in this process are ERCC1, ERCC2, ERCC3, ERCC4, ERCC5 and XPA. Previous studies have shown that the level of expression of these genes is important in the response and toxicity to platinum-based chemotherapy^26,27^. BER mechanism is responsible for eliminating base lesions in the genome, such as those caused by platinum-based chemotherapy or radiotherapy. The most studied genes in this mechanism are XRCC1 and XRCC2, and studies have associated the relationship between polymorphisms at this level with survival or response to chemotherapy^28^. Hence, in 161 cases, Sullivan et al. only found an association between the XRCC1 rs3213239 polymorphism and specific survival of patients with stage IV LC^14^. Among stage-IV patients in our study, in contrast, we only have found an association between 5-year survival and the AAT gene (MZ) polymorphism. In 137 LC cases in advanced stages (III/IV) treated with platinum-gemcitabine, Joerger et al. analyzed the association between 23 polymorphisms and survival and reported an association between ERCC1 rs11615 polymorphism (T allele) and shorter survival, when compared to patients with the C/C genotype^29^. However, the presence of this ERCC1 polymorphism (albeit in a different variant: rs3212986) in our study was associated with longer survival. This discordance could likely be due to the fact that the type of patients analyzed in our study were different (exclusively never-smoker subjects, all stages at diagnosis, higher percentage of women, etc.). In line with our findings, Ren et al. observed that the presence of ERCC1 rs11615 polymorphism (C/T or T/T) was associated with longer survival (*p* = 0.014)^30^. Furthermore, Ramos et al. in a recent systematic review, analyzed the association between GSTM1 and GSTT1 deletion and survival, concluding that most studies showed no effect or a slight trend towards a worse survival^13^. As can be seen, the results reported by these papers cited are heterogeneous. The majority of these studies have been conducted on LC patients -smokers as well as nonsmokers- among whom the latter tend to represent only a small percentage, so that the relationship between polymorphisms and survival in never-smokers is mostly unknown.

Toxicity due to treatment of LC patients has been associated with different genetic polymorphisms in the different studies published. For instance, Sullivan et al. reported an association between the ERCC2 (rs1799793) polymorphism and radiation pneumonitis^14^. In our study, however, the ERCC1 (rs11615) polymorphism was associated with toxicity in patients treated with chemotherapy plus radiotherapy. Yet in patients treated with platinum-based on chemotherapy, Ludovini et al. found no relationship, between the ERCC1, XRCC3 and XDP polymorphisms and toxicity^31^. In the case of the GSTT1 gene, Kalikaki et al. observed that it was associated with a higher risk of toxicity (76% of patients developed neutropenia vs. 24% of patients who did not have this polymorphism)^32^. In our study, in contrast, absence of the GSTT1 gene was associated with a different toxicity (nausea and vomiting). It should be noted: firstly, that none of the above studies specifically evaluated toxicity in never-smokers; and secondly, that polymorphisms can influence toxicity as well as response to treatment^33^.

There are likely differences in the relationship between genetic polymorphism and survival for smokers vs. never-smokers. The fundamental problem lies in the fact that there are few studies which specifically analyze the influence of polymorphisms among never-smokers. One example of these is Yang et al.’s study, which observed that the GSTP1 polymorphism was only associated with better survival in never-smoker patients^34^. LC in never-smokers is probably a different clinical entity (higher percentage of women, greater number of adenocarcinomas, greater presence of mutations in driver genes, etc.,) than that occurring in ever smokers, making it feasible that polymorphisms play a different role in never-smokers to that seen in smokers.

Similarly, it is known that the GSTM1 gene is implicated in the detoxification of metabolites of polycyclic aromatic hydrocarbons and that the GSTT1 gene is associated with the metabolism of other components of tobacco. This could be one reason why no association with survival is observed in never-smokers. GSTM1 and/or GSTT1 deletion produces an absence of enzymatic activity with an enhanced effect of chemotherapy treatment, greater oxidative damage in tumor cells and ensuingly higher toxicity, causing an expected probable increase in survival^13^.

This study has some advantages: it is multicentric and hospital-based, which lends it external validity. It is one of few studies that analyze the relationship between LC survival and the abovementioned polymorphisms in never-smokers, adjusting the results for residential radon concentration. In addition, it has a relatively large sample size. Since data-collection was based on electronic medical records, there were negligible losses to follow-up, and given that all the patients belonged to a given geographic area (Galicia), there was homogeneity in the treatments administered.

This study also has some limitations. Firstly, it analyzes a high number of low-frequency genetic polymorphisms, thereby reducing the study’s statistical power. Furthermore, the patients included few males (21%), most patients presented with stage IV at diagnosis (65%), and the majority of cancers were adenocarcinomas (83%), so that comparison with the rest of the literature published to date should be approached with caution. Secondly, we reported toxicity data according to electronical medical records and not to Common Toxicity Criteria. Finally, not all genes involved in BER and NER pathways have been analyzed and therefore a total picture on the involvement of these DNA repairing mechanisms could not be obtained.

In conclusion, survival at 5 years of diagnosis among never-smoker patients with LC was not observed to be associated with presence of genetic polymorphisms in genes that participate in the BER and NER pathways, except in the subgroup of patients who received radiotherapy or combined chemo-radiotherapy and presented with the ERCC1 rs3212986 polymorphism. Among the patients who received these treatments, higher toxicity was also found in the presence of OGG1 rs1052133 (CG) and ERCC1 rs11615 polymorphisms. Further studies are called for to elucidate the mechanisms whereby genetic polymorphisms can influence toxicity or survival among never-smoker LC patients.

## Supplementary information

Supplementary Information.
