# Supplementary material for: Polymorphisms in the BER and NER pathways and their influence on survival and toxicity in never-smokers with lung cancer
Source: Sci Rep. 2020 Dec 3;10:21147. doi: 10.1038/s41598-020-78051-5 (PMC7713126; doi:10.1038/s41598-020-78051-5)
Supplement: Supplementary file 1 — Supplementary Information. [file 41598_2020_78051_MOESM1_ESM.docx]

**Polymorphisms in the BER and NER Pathways and their Influence on Survival and Toxicity in Never-Smokers with Lung Cancer.**

**Authors**

Ana Casal-Mouriño,^a,b^ Alberto Ruano-Ravina,^b,c^ María Torres-Durán,^d^ Isaura Parente-Lamelas,^e^ Mariano Provencio-Pulla,^f^ Olalla Castro-Añón,^g^ Iria Vidal-García,^h^ José Abal-Arca,^e^ María Piñeiro-Lamas,^c^ Alberto Fernández-Villar,^d^ Luis Valdés-Cuadrado,^a,i^ Juan Miguel Barros-Dios,^b,c,j^ Mónica Pérez-Ríos.^b,c^

**Author affiliations**

^a^Department of Pneumology, Santiago de Compostela University Clinical Teaching Hospital, Santiago de Compostela, Spain

^b^Department of Preventive Medicine and Public Health, University of Santiago de Compostela, Santiago de Compostela, Spain

^c^Consortium for Biomedical Research in Epidemiology & Public Health (*CIBER en Epidemiología and Salud Pública - CIBERESP*), Spain

^d^Department of Pneumology, Vigo University Teaching Hospital Complex, Vigo, Spain

^e^Department of Pneumology, Ourense University Teaching Hospital Complex, Ourense, Spain

^f^Department of Oncology, Puerta de Hierro University Teaching Hospital, Madrid, Spain

^g^ Department of Pneumology, Lucus Augusti Hospital, Lugo, Spain

^h^ Department of Pneumology, A Coruña University Teaching Hospital Complex, Spain

^i^Interdisciplinary Neumology Research Group, Health Research Institute of Santiago de Compostela **(***Instituto Investigación Sanitaria de Santiago de Compostela/IDIS*), Spain.

^j^Department of Preventive Medicine, Santiago de Compostela University Teaching Hospital Complex, Santiago de Compostela, Spain.

**Address for correspondence:** Alberto Ruano-Ravina, Departamento de Medicina Preventiva y Salud Pública, Universidad de Medicina, C/San Francisco s/n, University of Santiago de Compostela, 15782. Santiago de Compostela, Spain. E-mail: [alberto.ruano@usc.es](mailto:alberto.ruano@usc.es)

**Contributions:** ACM and ARR have contributed to the conception and design of the study. ACM and MTD have contributed to acquisition, analysis and interpretation of data. All authors have revised the intellectual content, and have approved the final version to be submitted.

**Word count**

Abstract: 180

Text: 3,076

Tables: 8: 4 (current manuscript) and 4 (supplemental material)

Figures: 2

**Conflict of interests**

The authors confirm that there are no known conflicts of interest associated with this publication.

***This paper forms part of the work leading to Ana Casal-Mouriño’s PhD degree.***

**Supplemental information**

**Table S1. Survival at 3 years of diagnosis, according to treatment received and type of polymorphism.**

|  | **Chemotherapy** (179 patients and 121 events) | | | | **Other treatments** (112 patients and 64 events) | | | |
| --- | --- | --- | --- | --- | --- | --- | --- | --- |
| **Variable** | **Crude HR (IC 95%)** | **p-value** | **Adjusted HR^a^ (95% CI)** | **p-value** | **Crude HR (95% CI)** | **p-value** | **Adjusted HR^a^ (95% CI)** | **p-value** |
| **GSTM1** |  |  |  |  |  |  |  |  |
| Salvage gene/heterozygous | 1(-) |  | 1(-) |  | 1(-) |  | 1(-) |  |
| Homozygous | 1.1 (0.68-1.78) | 0,70 | 1.17 (0.72-1.9) | 0,52 | 0.59 (0.3-1.16) | 0,13 | 0.77 (0.33-1.79) | 0,54 |
| **GSTT1** |  |  |  |  |  |  |  |  |
| Salvage gene/heterozygous | 1(-) |  | 1(-) |  | 1(-) |  | 1(-) |  |
| Homozygous | 1.29 (0.76-2.19) | 0,35 | 1.29 (0.75-2.2) | 0,35 | 0.83 (0.36-1.91) | 0,67 | 1.07 (0.42-2.72) | 0,88 |
| **XRCC1 rs25487** |  |  |  |  |  |  |  |  |
| GG | 1(-) |  | 1(-) |  | 1(-) |  | 1(-) |  |
| AG | 1.27 ( 0.83 - 1.93 ) | 0,27 | 1.23 ( 0.8 - 1.87 ) | 0,35 | 1.08 ( 0.61 - 1.9 ) | 0,88 | 1.59 ( 0.85 - 2.99 ) | 0,19 |
| AA | 1.2 ( 0.67 - 2.15 ) | 0,53 | 1.48 ( 0.81 - 2.7 ) | 0,20 | 0.88 ( 0.31 - 2.5 ) | 0,76 | 1.18 ( 0.34 - 4.06 ) | 0,85 |
| **ERCC1 rs11615** |  |  |  |  |  |  |  |  |
| CC | 1(-) |  | 1(-) |  | 1(-) |  | 1(-) |  |
| CT | 0.93 ( 0.56 - 1.57 ) | 0,79 | 1.09 ( 0.64 - 1.83 ) | 0,76 | 0.58 ( 0.29 - 1.19 ) | 0,14 | 0.73 ( 0.34 - 1.59 ) | 0,46 |
| TT | 0.56 ( 0.31 - 1.01 ) | 0,06 | 0.71 ( 0.39 - 1.29 ) | 0,27 | 0.62 ( 0.29 - 1.32 ) | 0,27 | 0.79 ( 0.36 - 1.75 ) | 0,67 |
| **ERCC1 rs3212986** |  |  |  |  |  |  |  |  |
| GG | 1(-) |  | 1(-) |  | 1(-) |  | 1(-) |  |
| GT | 1.41 ( 0.94 - 2.11 ) | 0,09 | 1.21 ( 0.8 - 1.84 ) | 0,36 | 1.24 ( 0.7 - 2.21 ) | 0,54 | 1.05 ( 0.57 - 1.92 ) | 0,98 |
| TT | 1.65 ( 0.81 - 3.37 ) | 0,17 | 1.56 ( 0.76 - 3.22 ) | 0,22 | 3.72 ( 1.59 - 8.71 ) | **<0,001** | 3.48 ( 1.39 - 8.69 ) | **0,01** |
| **ERCC2 rs13181** |  |  |  |  |  |  |  |  |
| TT | 1(-) |  | 1(-) |  | 1(-) |  | 1(-) |  |
| GT | 0.95 ( 0.63 - 1.42 ) | 0,79 | 1.08 ( 0.71 - 1.63 ) | 0,72 | 0.73 ( 0.4 - 1.32 ) | 0,37 | 1.1 ( 0.57 - 2.12 ) | 0,62 |
| GG | 1.2 ( 0.63 - 2.26 ) | 0,58 | 1.13 ( 0.6 - 2.15 ) | 0,71 | 1.6 ( 0.75 - 3.42 ) | 0,23 | 2 ( 0.87 - 4.58 ) | 0,10 |
| **XRCC3 rs861539** |  |  |  |  |  |  |  |  |
| CC | 1(-) |  | 1(-) |  | 1(-) |  | 1(-) |  |
| TC | 0.91 ( 0.6 - 1.39 ) | 0,68 | 1 ( 0.66 - 1.53 ) | 0,99 | 0.93 ( 0.53 - 1.64 ) | 0,90 | 1.09 ( 0.59 - 2 ) | 0,69 |
| TT | 0.65 ( 0.35 - 1.2 ) | 0,17 | 0.87 ( 0.46 - 1.62 ) | 0,65 | 1.08 ( 0.44 - 2.66 ) | 0,87 | 1.24 ( 0.5 - 3.08 ) | 0,65 |
| **OGG1 rs1052133** |  |  |  |  |  |  |  |  |
| CC | 1(-) |  | 1(-) |  | 1(-) |  | 1(-) |  |
| CG | 1 ( 0.66 - 1.51 ) | 0,99 | 0.95 ( 0.63 - 1.44 ) | 0,82 | 0.69 ( 0.37 - 1.27 ) | 0,20 | 0.47 ( 0.24 - 0.91 ) | **0,02** |
| GG | 1.94 ( 0.84 - 4.48 ) | 0,12 | 1.42 ( 0.6 - 3.35 ) | 0,42 | 3.3 ( 1 - 10.91 ) | 0,06 | 1.27 ( 0.38 - 4.26 ) | 0,74 |
| **AAT** |  |  |  |  |  |  |  |  |
| MM | 1(-) |  | 1(-) |  | 1(-) |  | 1(-) |  |
| MS | 1.11 ( 0.71 - 1.74 ) | 0,64 | 0.98 ( 0.61 - 1.57 ) | 0,93 | 1.43 ( 0.76 - 2.69 ) | 0,31 | 0.97 ( 0.49 - 1.92 ) | 0,81 |
| MZ | 0.81 ( 0.11 - 5.82 ) | 0,83 | 1.18 ( 0.16 - 8.88 ) | 0,87 | 1.5 ( 0.36 - 6.22 ) | 0,60 | 1.58 ( 0.34 - 7.27 ) | 0,55 |
| SZ | 5.57 ( 0.75 - 41.22 ) | 0,09 | 3.5 ( 0.46 - 26.7 ) | 0,23 | 2.02 ( 0.28 - 14.81 ) | 0,51 | 2.21 ( 0.26 - 18.48 ) | 0,44 |
| SS | 0.42 ( 0.06 - 3.01 ) | 0,39 | 0.46 ( 0.06 - 3.37 ) | 0,45 | 0.86 ( 0.12 - 6.25 ) | 0,86 | 4.38 ( 0.51 - 38 ) | 0,18 |

^a^ Adjusted for age, sex, environmental tobacco smoke, and indoor radon exposure.

**Table S2. Survival at 3 years, according to the different polymorphisms and treatment received for patients in Stage IV.**

|  | **Chemotherapy** (119 patients and 94 events) | | | | **Other treatments** (58 patients and 43 events) | | | |
| --- | --- | --- | --- | --- | --- | --- | --- | --- |
| **Variable** | **Crude HR (95% CI)** | **p-value** | **Adjusted HR^a^ (95% CI)** | **p-value** | **Crude HR (95% CI)** | **p-value** | **Adjusted HR^a^ (95% CI)** | **p-value** |
| **GSTM1** |  |  |  |  |  |  |  |  |
| Salvage gene/heterozygous | 1(-) |  | 1(-) |  | 1(-) |  | 1(-) |  |
| Homozygous | 1.27 ( 0.74 - 2.16 ) | 0,39 | 1.3 ( 0.76 - 2.23 ) | 0,33 | 1.06 ( 0.47 - 2.41 ) | 0,899 | 1.17 ( 0.45 - 3.02 ) | 0,75 |
| **GSTT1** |  |  |  |  |  |  |  |  |
| Salvage gene/heterozygous | 1(-) |  | 1(-) |  | 1(-) |  | 1(-) |  |
| Homozygous | 1.24 ( 0.68 - 2.24 ) | 0,49 | 1.24 ( 0.68 - 2.25 ) | 0,48 | 1.04 ( 0.41 - 2.65 ) | 0,93 | 1.3 ( 0.46 - 3.73 ) | 0,62 |
| **XRCC1 rs25487** |  |  |  |  |  |  |  |  |
| GG | 1(-) |  | 1(-) |  | 1(-) |  | 1(-) |  |
| AG | 1.16 ( 0.72 - 1.86 ) | 0,54 | 1.15 ( 0.72 - 1.84 ) | 0,57 | 1.22 ( 0.62 - 2.43 ) | 0,57 | 1.47 ( 0.69 - 3.15 ) | 0,32 |
| AA | 1.74 ( 0.88 - 3.45 ) | 0,11 | 1.63 ( 0.81 - 3.28 ) | 0,17 | 3.2 ( 1.06 - 9.68 ) | **0,04** | 2.23 ( 0.59 - 8.38 ) | 0,24 |
| **ERCC1 rs11615** |  |  |  |  |  |  |  |  |
| CC | 1(-) |  | 1(-) |  | 1(-) |  | 1(-) |  |
| CT | 0.98 ( 0.56 - 1.69 ) | 0,93 | 1 ( 0.58 - 1.73 ) | 0,99 | 0.74 ( 0.32 - 1.72 ) | 0,49 | 0.65 ( 0.26 - 1.62 ) | 0,35 |
| TT | 0.59 ( 0.3 - 1.13 ) | 0,11 | 0.6 ( 0.31 - 1.16 ) | 0,13 | 1.02 ( 0.44 - 2.34 ) | 0,97 | 0.97 ( 0.41 - 2.28 ) | 0,95 |
| **ERCC1 rs3212986** |  |  |  |  |  |  |  |  |
| GG | 1(-) |  | 1(-) |  | 1(-) |  | 1(-) |  |
| GT | 1.32 ( 0.83 - 2.08 ) | 0,24 | 1.27 ( 0.79 - 2.02 ) | 0,32 | 1.15 ( 0.58 - 2.28 ) | 0,68 | 0.98 ( 0.49 - 1.98 ) | 0,97 |
| TT | 1.65 ( 0.73 - 3.75 ) | 0,23 | 1.62 ( 0.71 - 3.69 ) | 0,25 | 1.9 ( 0.63 - 5.72 ) | 0,25 | 2.93 ( 0.91 - 9.39 ) | 0,07 |
| **ERCC2 rs13181** |  |  |  |  |  |  |  |  |
| TT | 1(-) |  | 1(-) |  | 1(-) |  | 1(-) |  |
| GT | 1.05 ( 0.66 - 1.68 ) | 0,84 | 1.05 ( 0.66 - 1.69 ) | 0,83 | 0.78 ( 0.38 - 1.6 ) | 0,50 | 1.02 ( 0.49 - 2.15 ) | 0,96 |
| GG | 1.18 ( 0.6 - 2.31 ) | 0,63 | 1.17 ( 0.6 - 2.3 ) | 0,65 | 1.08 ( 0.45 - 2.58 ) | 0,87 | 1.61 ( 0.64 - 4.06 ) | 0,31 |
| **XRCC3 rs861539** |  |  |  |  |  |  |  |  |
| CC | 1(-) |  | 1(-) |  | 1(-) |  | 1(-) |  |
| TC | 0.92 ( 0.58 - 1.45 ) | 0,71 | 0.92 ( 0.58 - 1.46 ) | 0,73 | 1.17 ( 0.6 - 2.28 ) | 0,64 | 1.24 ( 0.63 - 2.46 ) | 0,53 |
| TT | 0.66 ( 0.29 - 1.5 ) | 0,32 | 0.68 ( 0.3 - 1.55 ) | 0,36 | 1.11 ( 0.32 - 3.83 ) | 0,87 | 0.91 ( 0.26 - 3.18 ) | 0,88 |
| **OGG1 rs1052133** |  |  |  |  |  |  |  |  |
| CC | 1(-) |  | 1(-) |  | 1(-) |  | 1(-) |  |
| CG | 1.02 ( 0.64 - 1.62 ) | 0,94 | 1.01 ( 0.63 - 1.61 ) | 0,96 | 0.66 ( 0.31 - 1.41 ) | 0,29 | 0.51 ( 0.23 - 1.1 ) | 0,09 |
| GG | 1.52 ( 0.61 - 3.83 ) | 0,37 | 1.37 ( 0.53 - 3.52 ) | 0,51 | 1.67 ( 0.5 - 5.59 ) | 0,41 | 1.26 ( 0.37 - 4.26 ) | 0,71 |
| **AAT** |  |  |  |  |  |  |  |  |
| MM | 1(-) |  | 1(-) |  | 1(-) |  | 1(-) |  |
| MS | 0.99 ( 0.6 - 1.65 ) | 0,98 | 0.97 ( 0.57 - 1.64 ) | 0,90 | 1.46 ( 0.69 - 3.12 ) | 0,32 | 1.09 ( 0.5 - 2.4 ) | 0,83 |
| MZ | 7.51 ( 0.98 - 57.86 ) | 0,05 | 6.92 ( 0.86 - 55.76 ) | 0,07 | 0.62 ( 0.08 - 4.62 ) | 0,64 | 1.06 ( 0.13 - 8.46 ) | 0,96 |
| SZ | 4.5 ( 0.6 - 33.69 ) | 0,14 | 4.16 ( 0.53 - 32.47 ) | 0,17 | 0.95 ( 0.13 - 7.01 ) | 0,98 | 1.35 ( 0.16 - 11.08 ) | 0,78 |
| SS | 0.56 ( 0.08 - 4.03 ) |  | 0.58 ( 0.08 - 4.27 ) |  | -- |  | -- |  |

^a^ Adjusted for age, sex, environmental tobacco smoke, and indoor radon exposur

**Table S3. Toxicity for the different polymorphisms, according to treatment received (radiotherapy or chemo-radiotherapy).**

|  | **Radiotherapy toxicity** | | | | | | **Chemo-radiotherapy toxicity** | | | | | |
| --- | --- | --- | --- | --- | --- | --- | --- | --- | --- | --- | --- | --- |
| **Variable** | **Cases, n (%)** | **Controls, n (%)** | **Crude OR (95% CI)** | **p-value** | **Adjusted OR^a^ (95% CI)** | **p-value** | **Cases, n (%)** | **Controls, n(%)** | **Crude OR (95% CI)** | **p-value** | **Adjusted OR^a^ (95% CI)** | **p-value** |
| **GSTM1** |  |  |  |  |  |  |  |  |  |  |  |  |
| Salvage gene/heterozygous | 5 (71.4) | 9 (64.3) | 1(-) |  | 1(-) |  | 14 (58.3) | 10 (38.5) | 1(-) |  | 1(-) |  |
| Homozygous | 2 (28.6) | 5 (35.7) | 0.72 ( 0.08 - 4.9 ) | 0,74 | 0.74 ( 0.02 - 16.14 ) | 0,86 | 10 (41.7) | 16 (61.5) | 0.45 ( 0.14 - 1.37 ) | 0,16 | 0.74 ( 0.2 - 2.78 ) | 0,66 |
| **GSTT1** |  |  |  |  |  |  |  |  |  |  |  |  |
| Salvage gene/heterozygous | 4 (66.7) | 9 (69.2) | 1(-) |  | 1(-) |  | 21 (87.5) | 18 (75) | 1(-) |  | 1(-) |  |
| Homozygous | 2 (33.3) | 4 (30.8) | 1.12 ( 0.12 - 8.78 ) | 0,91 | 1.38 ( 0.07 - 43.86 ) | 0,83 | 3 (12.5) | 6 (25) | 0.43 ( 0.08 - 1.87 ) | 0,28 | 0.32 ( 0.05 - 1.74 ) | 0,21 |
| **XRCC1 rs25487** |  |  |  |  |  |  |  |  |  |  |  |  |
| GG | 7 (63.6) | 13 (52) | 1(-) |  | 1(-) |  | 21 (50) | 15 (34.1) | 1(-) |  | 1(-) |  |
| AG | 4 (36.4) | 10 (40) | 0.74 ( 0.16 - 3.2 ) | 0,69 | 0.12 ( 0.01 - 1.21 ) | 0,11 | 15 (35.7) | 23 (52.3) | 0.47 ( 0.18 - 1.17 ) | 0,11 | 0.46 ( 0.16 - 1.3 ) | 0,15 |
| AA | -- | 2 (8 ) | -- |  | -- |  | 6 (14.3) | 6 (13.6) | 0.71 ( 0.19 - 2.7 ) | 0,62 | 0.46 ( 0.09 - 2.16 ) | 0,33 |
| **ERCC1 rs11615** |  |  |  |  |  |  |  |  |  |  |  |  |
| CC | 2 (18.2) | 5 (20) | 1(-) |  | 1(-) |  | 3 (7.1) | 11 (25) | 1(-) |  | 1(-) |  |
| CT | 4 (36.4) | 10 (40) | 1 ( 0.14 - 9.04 ) | 1 | 1.52 ( 0.05 - 60.87 ) | 0,81 | 21 (50) | 23 (52.3) | 3.35 ( 0.9 - 16.32 ) | 0,09 | 6.29 ( 1.18 - 53.48 ) | **0,04** |
| TT | 5 (45.5) | 10 (40) | 1.25 ( 0.19 - 10.95 ) | 0,82 | 0.27 ( 0 - 10.83 ) | 0,48 | 18 (42.9) | 10 (22.7) | 6.6 ( 1.63 - 34.62 ) | **0,01** | 25.57 ( 3.7 - 281.65 ) | **0,01** |
| **ERCC1 rs3212986** |  |  |  |  |  |  |  |  |  |  |  |  |
| GG | 8 (72.7) | 16 (64) | 1(-) |  | 1(-) |  | 25 (59.5) | 17 (38.6) | 1(-) |  | 1(-) |  |
| GT | 3 (27.3) | 5 (20) | 1.2 ( 0.2 - 6.26 ) | 0,83 | 2.64 ( 0.17 - 74.31 ) | 0,50 | 13 (31) | 23 (52.3) | 0.38 ( 0.15 - 0.95 ) | **0,04** | 0.2 ( 0.06 - 0.64 ) | **0,01** |
| TT | -- | 4 (16) | -- |  | -- |  | 4 (9.5) | 4 (9.1) | 0.68 ( 0.14 - 3.23 ) | 0,62 | 0.36 ( 0.05 - 2.19 ) | 0,27 |
| **ERCC2 rs13181** |  |  |  |  |  |  |  |  |  |  |  |  |
| TT | 5 (45.5) | 10 (40) | 1(-) |  | 1(-) |  | 18 (42.9) | 19 (43.2) | 1(-) |  | 1(-) |  |
| GT | 5 (45.5) | 12 (48) | 1.2 ( 0.26 - 5.52 ) | 0,81 | 4.44 ( 0.48 - 76.46 ) | 0,23 | 19 (45.2) | 17 (38.6) | 0.85 ( 0.34 - 2.13 ) | 0,72 | 0.67 ( 0.22 - 1.93 ) | 0,46 |
| GG | 1 (9.1) | 3 (12) | 0.8 ( 0.03 - 8.21 ) | 0,86 | 1.97 ( 0 - 6354.24 ) | 0,89 | 5 (11.9) | 8 (18.2) | 0.56 ( 0.14 - 2.01 ) | 0,38 | 0.22 ( 0.04 - 1.1 ) | 0,08 |
| **XRCC3 rs861539** |  |  |  |  |  |  |  |  |  |  |  |  |
| CC | 5 (45.5) | 12 (48) | 1(-) |  | 1(-) |  | 9 (21.4) | 12 (27.3) | 1(-) |  | 1(-) |  |
| TC | 3 (27.3) | 10 (40) | 0.72 ( 0.12 - 3.71 ) | 0,70 | 2.42 ( 0.13 - 100.23 ) | 0,57 | 24 (57.1) | 27 (61.4) | 1.19 ( 0.43 - 3.37 ) | 0,7451 | 1.22 ( 0.38 - 4.05 ) | 0,7393 |
| TT | 3 (27.3) | 3 (12) | 2.4 ( 0.34 - 17.52 ) | 0,37 | 41.8 ( 1.42 - 4528.11 ) | 0,06 | 9 (21.4) | 5 (11.4) | 2.4 ( 0.61 - 10.28 ) | 0,2182 | 2.57 ( 0.54 - 13.73 ) | 0,2481 |
| **OGG1 rs1052133** |  |  |  |  |  |  |  |  |  |  |  |  |
| CC | 4 (36.4) | 19 (76) | 1(-) |  | 1(-) |  | 28 (66.7) | 26 (59.1) | 1(-) |  | 1(-) |  |
| CG | 7 (63.6) | 5 (20) | 6.65 ( 1.45 - 35.65 ) | **0,02** | -- |  | 12 (28.6) | 16 (36.4) | 0.7 ( 0.27 - 1.74 ) | 0,44 | 0.48 ( 0.16 - 1.34 ) | 0,17 |
| GG | -- | 1 (4) | -- |  | -- |  | 2 (4.8) | 2 (4.5) | 0.93 ( 0.11 - 8.2 ) | 0,94 | 1.11 ( 0.11 - 11.47 ) | 0,93 |
| **Alpha-1-antytripsin** |  |  |  |  |  |  |  |  |  |  |  |  |
| MM | 9 (81.8) | 17 (68) | 1(-) |  | 1(-) |  | 33 (80.5) | 34 (77.3) | 1(-) |  | 1(-) |  |
| MS | 2 (18.2) | 4 (16) | 0.94 ( 0.11 - 5.88 ) | 0,95 | 1.82 ( 0.04 - 88.49 ) | 0,74 | 7 (17.1) | 9 (20.5) | 0.8 ( 0.26 - 2.4 ) | 0,69 | 1.09 ( 0.32 - 3.78 ) | 0,89 |
| MZ | -- | 3 (12) | -- |  | -- |  | -- | 1 (2.3) | -- |  | -- |  |
| SZ | -- | -- | -- |  | -- |  | -- | -- | -- |  | -- |  |
| SS | -- | 1 (4) | -- |  | -- |  | 1 (2.4) | -- | -- |  | -- |  |

^a^ Adjusted for age, sex, environmental tobacco smoke, indoor radon exposure, and stage at diagnosis.

**Table S4. Most frequent toxicities found, according to presence of genetic polymorphisms.**

|  | **Toxicity: anti-EGFR toxicoderma** | | | **Toxicity: Nausea/ vomiting** | | |
| --- | --- | --- | --- | --- | --- | --- |
| **Variable** | **No, n (%)** | **Yes, n (%)** | ***p*-value** | **No, n (%)** | **Yes, n (%)** | ***p*-value** |
| **GSTM1** |  |  |  |  |  |  |
| Salvage gene/heterozygous | 68 (81.9) | 15 (18.1) | 0.41 | 79 (95.2) | 4 (4.8) | 0.43 |
| Homozygous | 80 (87) | 12 (13) |  | 90 (97.8) | 2 (2.2) |  |
| **GSTT1** |  |  |  |  |  |  |
| Salvage gene/heterozygous | 103 (82.4) | 22 (17.6) | 0.47 | 120 (96) | 5 (4) | 1 |
| Homozygous | 39 (88.6) | 5 (11.4) |  | 42 (95.5) | 2 (4.5) |  |
| **XRCC1 rs25487** |  |  |  |  |  |  |
| GG | 123 (82.6) | 26 (17.4) | 0.26 | 142 (95.3) | 7 (4.7) | 0.85 |
| AG | 111 (86) | 18 (14) |  | 121 (93.8) | 8 (6.2) |  |
| AA | 38 (92.7) | 3 (7.3) |  | 39 (95.1) | 2 (4.9) |  |
| **ERCC1 rs11615** |  |  |  |  |  |  |
| CC | 47 (87) | 7 (13) | 0.90 | 52 (96.3) | 2 (3.7) | 0.75 |
| CT | 134 (85.4) | 23 (14.6) |  | 149 (94.9) | 8 (5.1) |  |
| TT | 91 (84.3) | 17 (15.7) |  | 101 (93.5) | 7 (6.5) |  |
| **ERCC1 rs3212986** |  |  |  |  |  |  |
| GG | 147 (86) | 24 (14) | 0.87 | 163 (95.3) | 8 (4.7) | 0.81 |
| GT | 107 (84.9) | 19 (15.1) |  | 118 (93.7) | 8 (6.3) |  |
| TT | 18 (81.8) | 4 (18.2) |  | 21 (95.5) | 1 (4.5) |  |
| **ERCC2 rs13181** |  |  |  |  |  |  |
| TT | 114 (84.4) | 21 (15.6) | 0.84 | 128 (94.8) | 7 (5.2) | 0.73 |
| GT | 128 (86.5) | 20 (13.5) |  | 139 (93.9) | 9 (6.1) |  |
| GG | 30 (83.3) | 6 (16.7) |  | 35 (97.2) | 1 (2.8) |  |
| **XRCC3 rs861539** |  |  |  |  |  |  |
| CC | 100 (87.7) | 14 (12.3) | 0.64 | 111 (97.4) | 3 (2.6) | 0.23 |
| TC | 138 (84.1) | 26 (15.9) |  | 152 (92.7) | 12 (7.3) |  |
| TT | 34 (82.9) | 7 (17.1) |  | 39 (95.1) | 2 (4.9) |  |
| **OGG1 rs1052133** |  |  |  |  |  |  |
| CC | 166 (84.7) | 30 (15.3) | 0.57 | 185 (94.4) | 11 (5.6) | Not possible to apply Chi-square test |
| CG | 90 (84.9) | 16 (15.1) |  | 100 (94.3) | 6 (5.7) |  |
| GG | 16 (94.1) | 1 (5.9) |  | 17 (100) | -- |  |
| **Alpha-1-antytripsin** |  |  |  |  |  |  |
| MM | 196 (84.8) | 35 (15.2) | Not possible to apply Chi-square test | 216 (93.5) | 15 (6.5) | Not possible to apply Chi-square test |
| MS | 59 (88.1) | 8 (11.9) |  | 65 (97) | 2 (3) |  |
| MZ | 8 (88.9) | 1 (11.1) |  | 9 (100) | -- |  |
| SZ | 3 (100) | -- |  | 3 (100) | -- |  |
| SS | 6 (85.7) | 1 (14.3) |  | 7 (100) | -- |  |

**Figure 1. 5-year lung cancer survival according to the treatment received and polymorphisms analyzed or mutation status: A) Chemotherapy – GSTM1, B) Chemotherapy – GSTT1; C) Other treatments – GSTM1; D) Other treatments – GSTT1; E) Chemotherapy – EGFR; F) Chemotherapy – ALK; G) Other treatments – EGFR; H) Other treatments – ALK.**

**
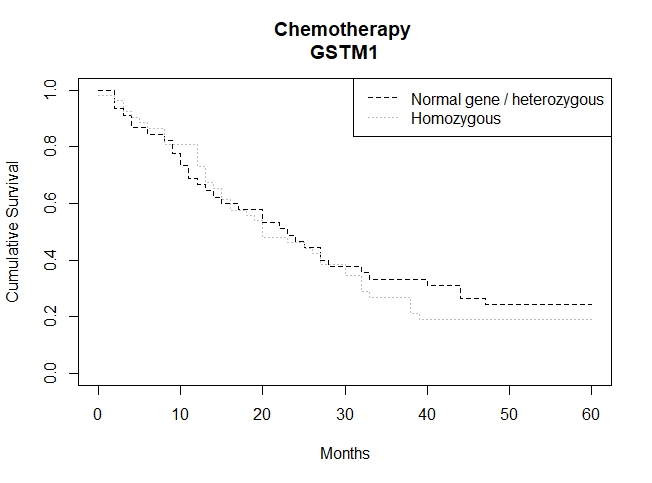

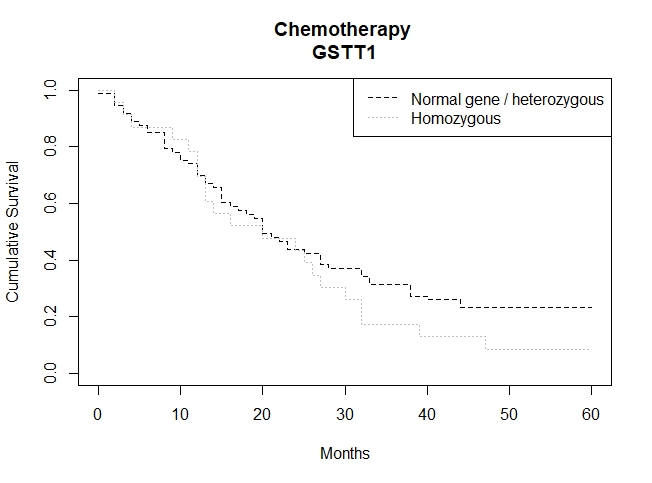

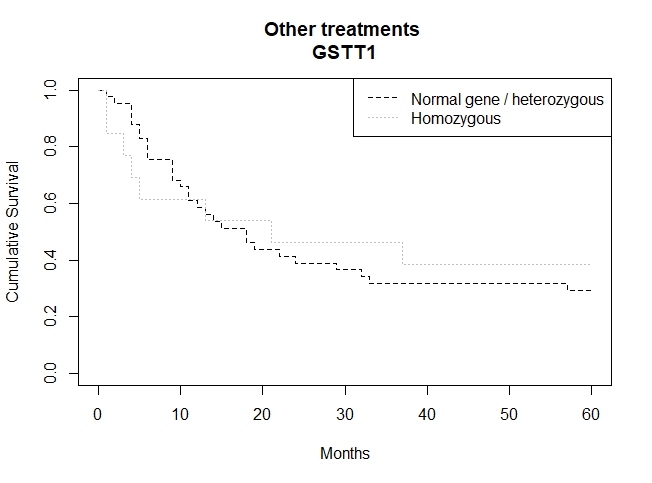

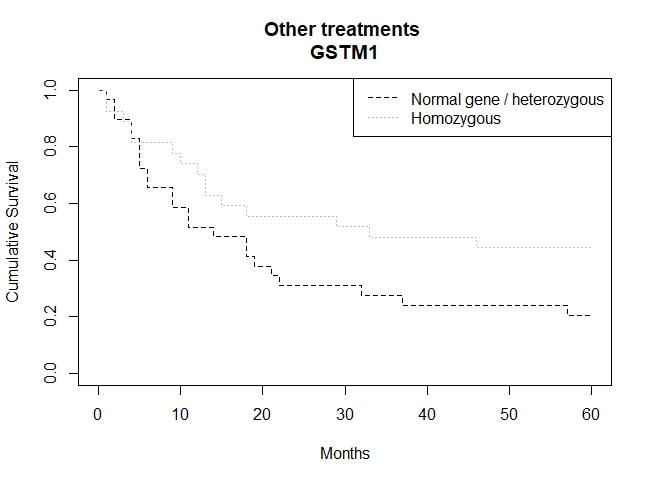
**

**B)**

**C)**

**D)**

**A)**

**
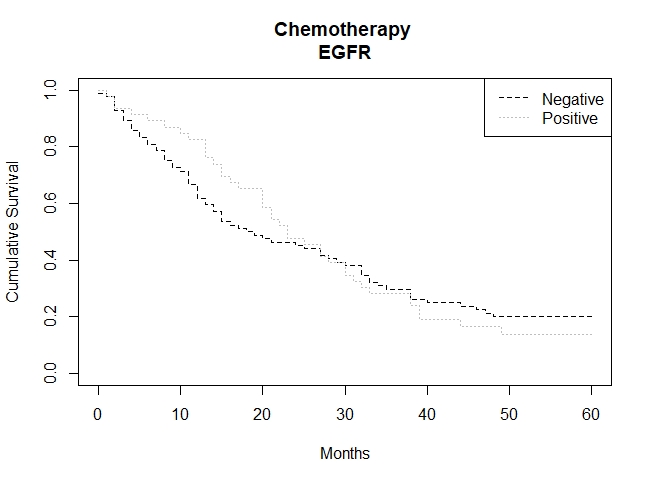

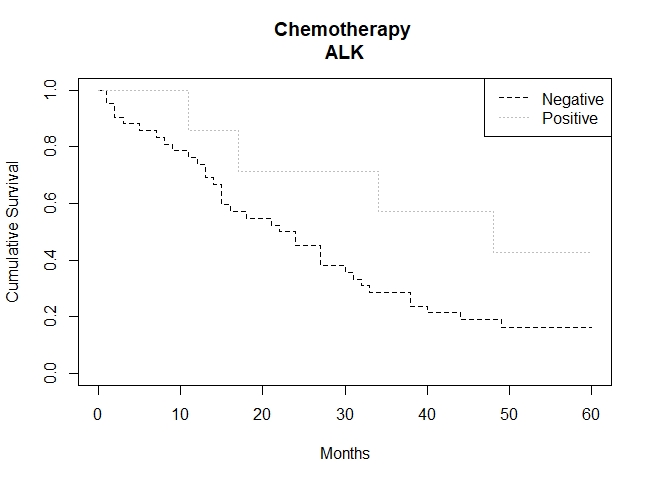

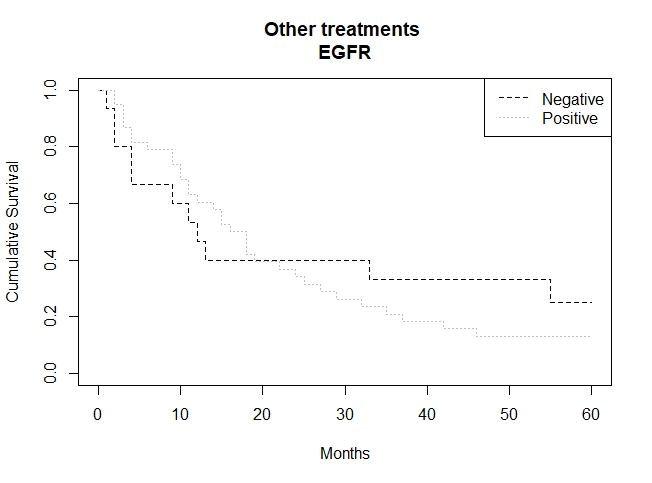

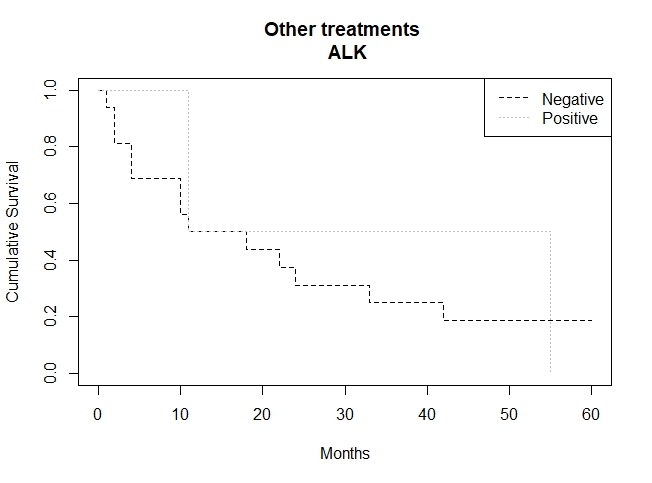
**

**E)** **
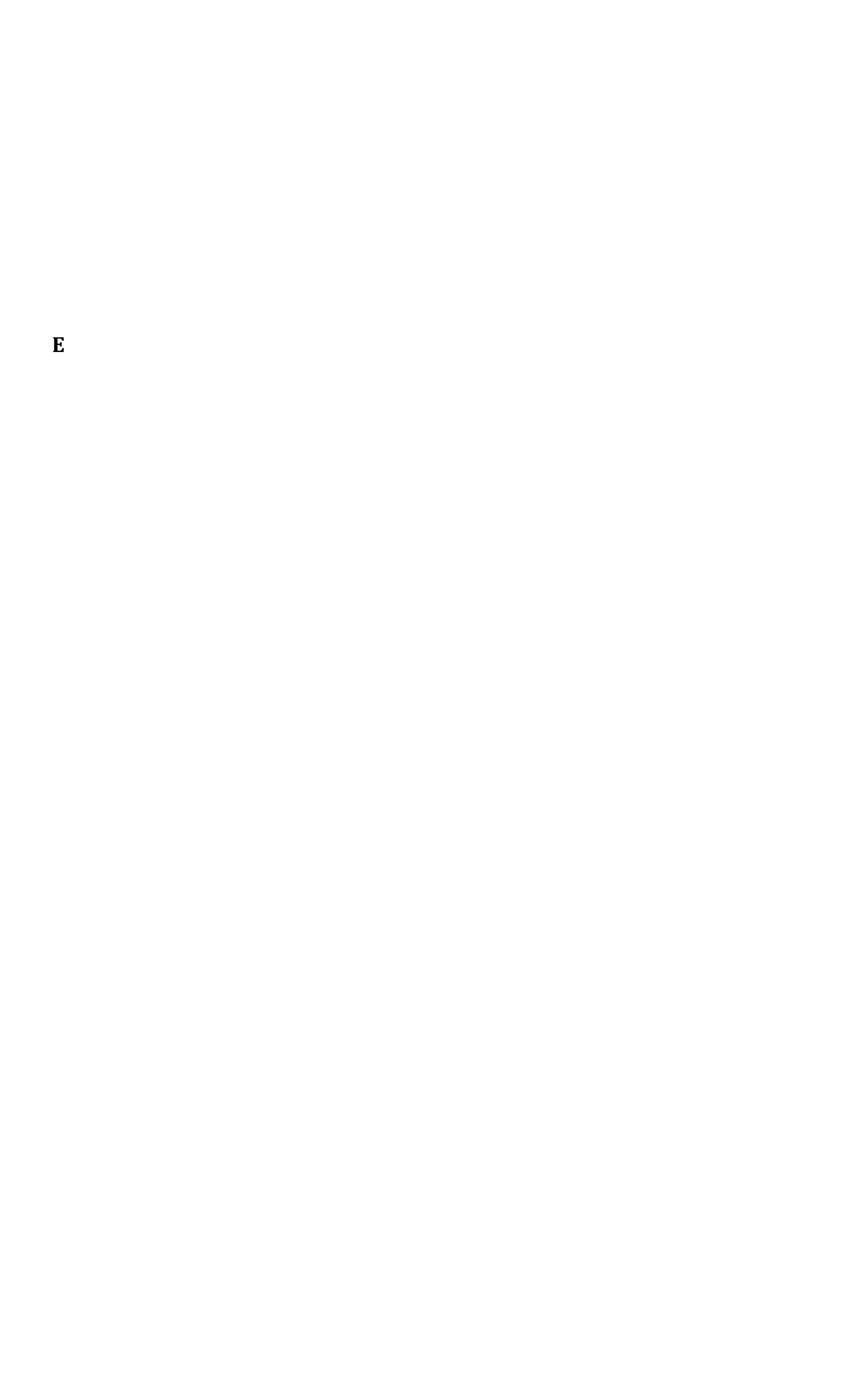

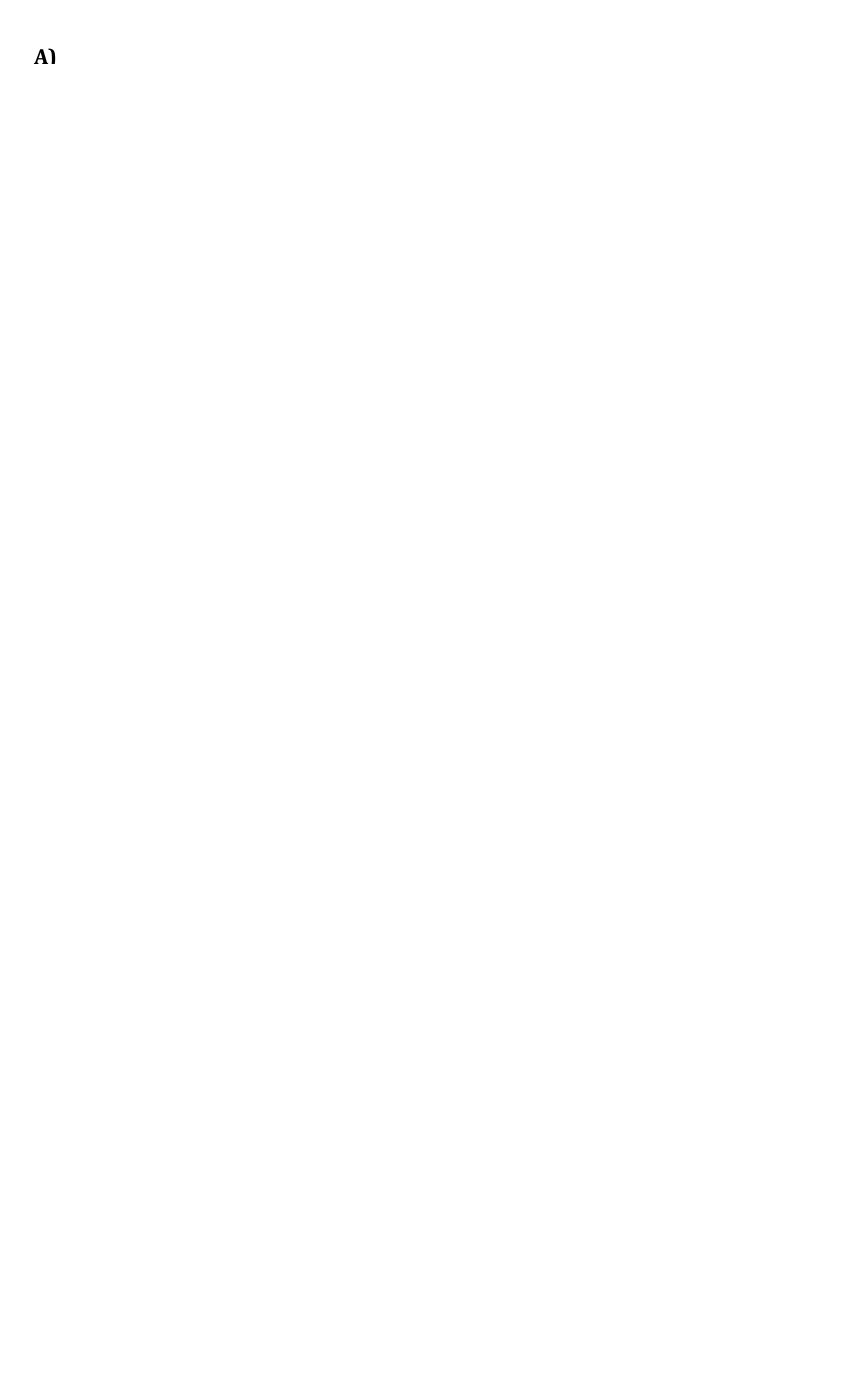
)**

**F)** **
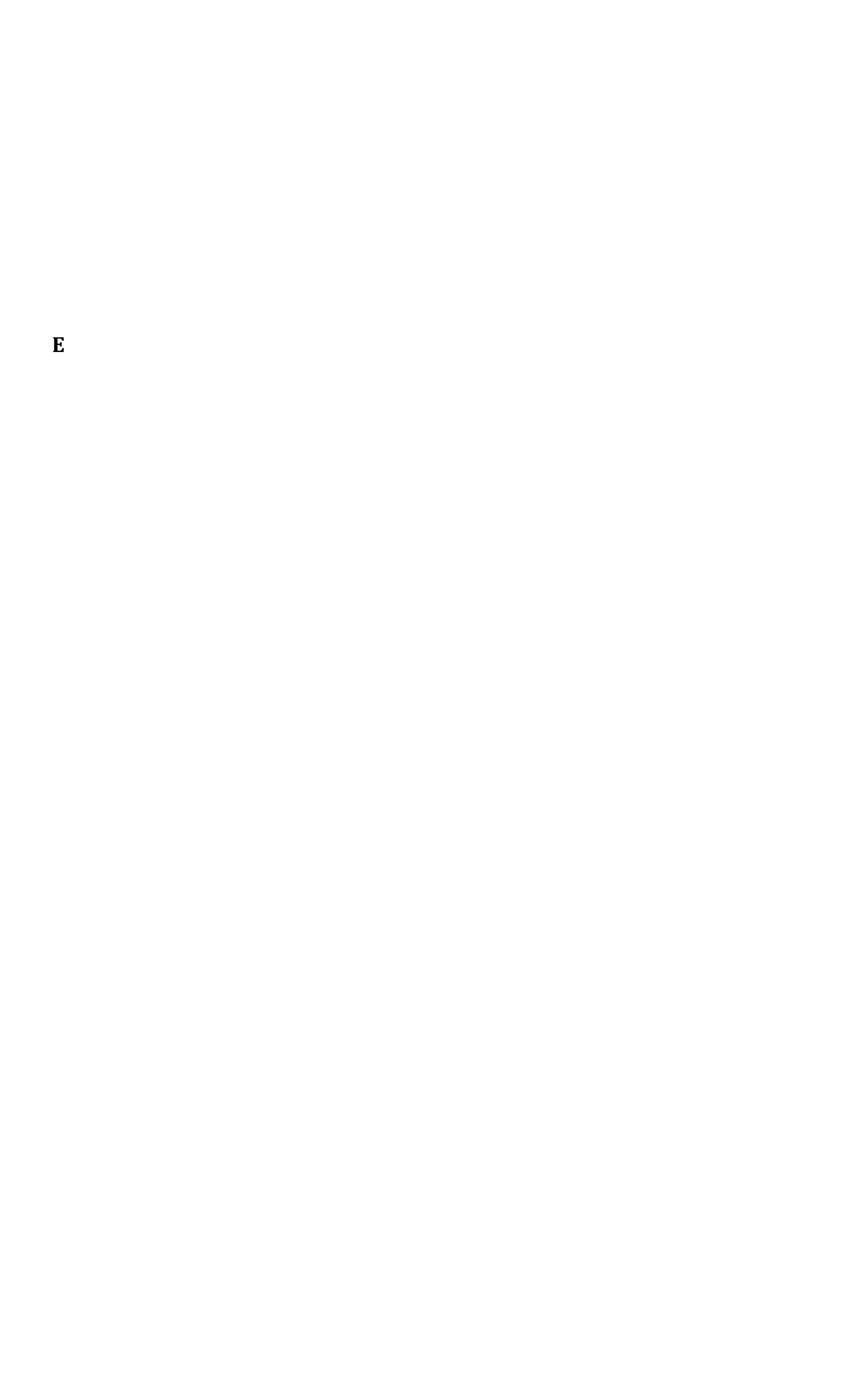

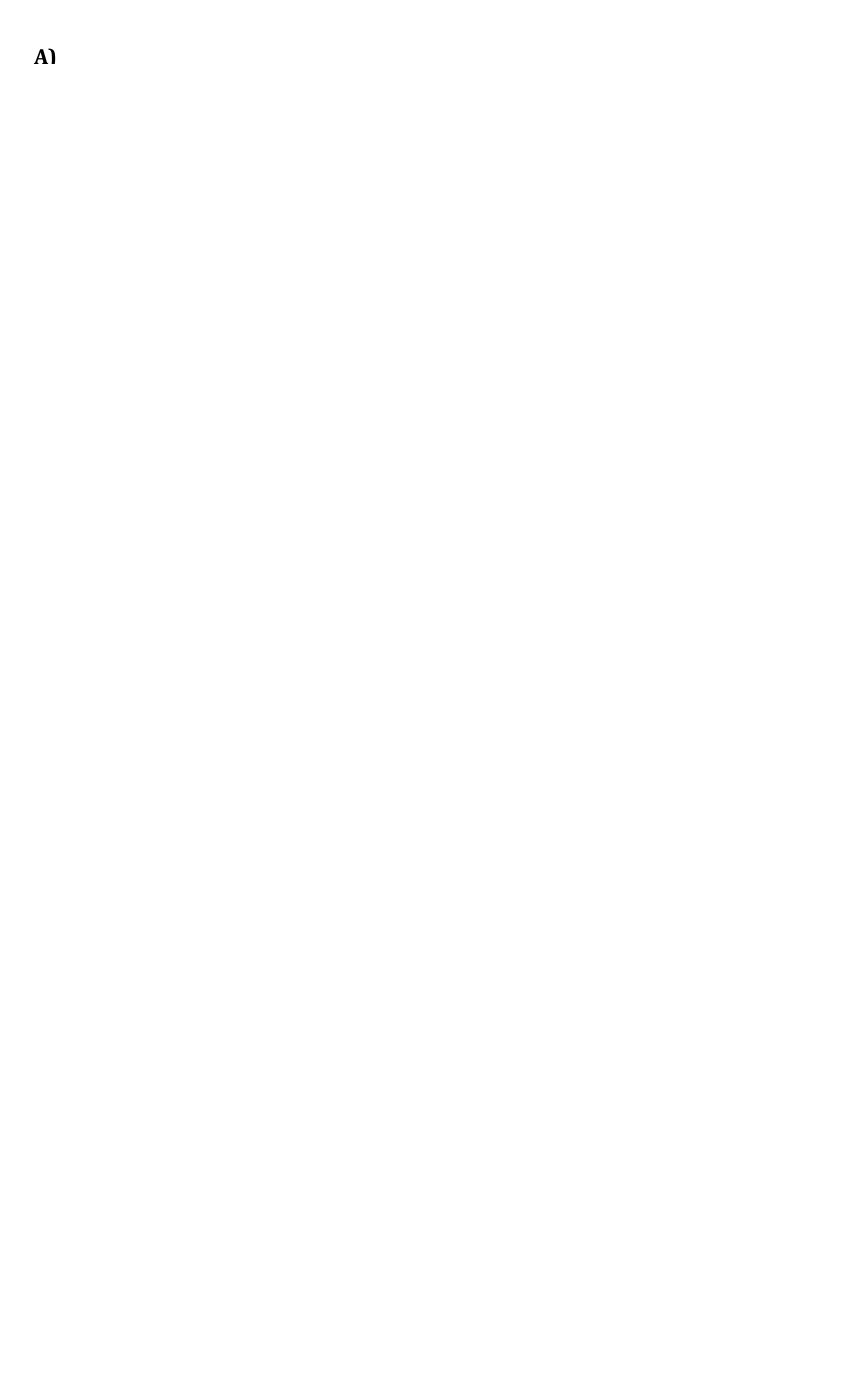
)**

**G)** **
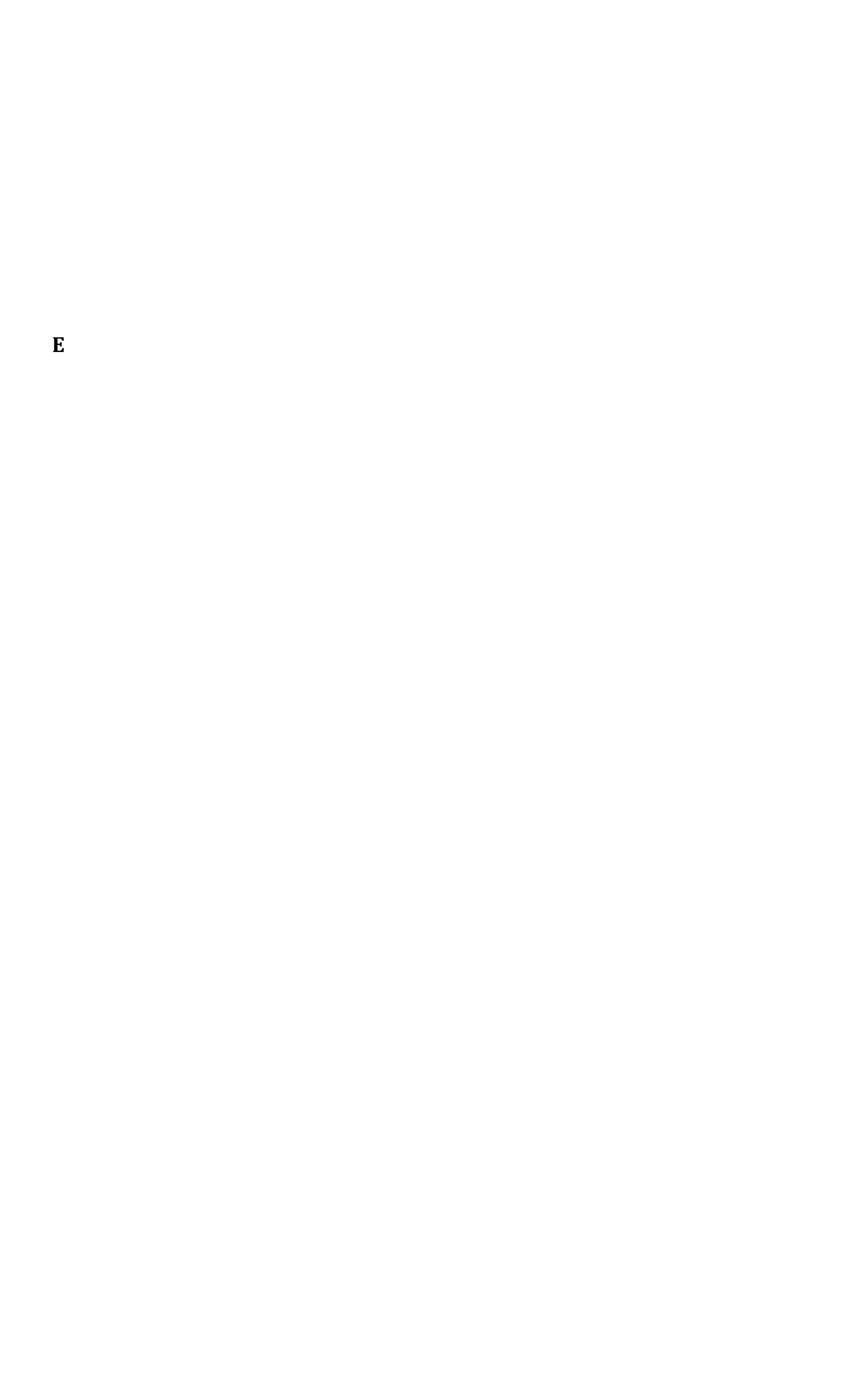

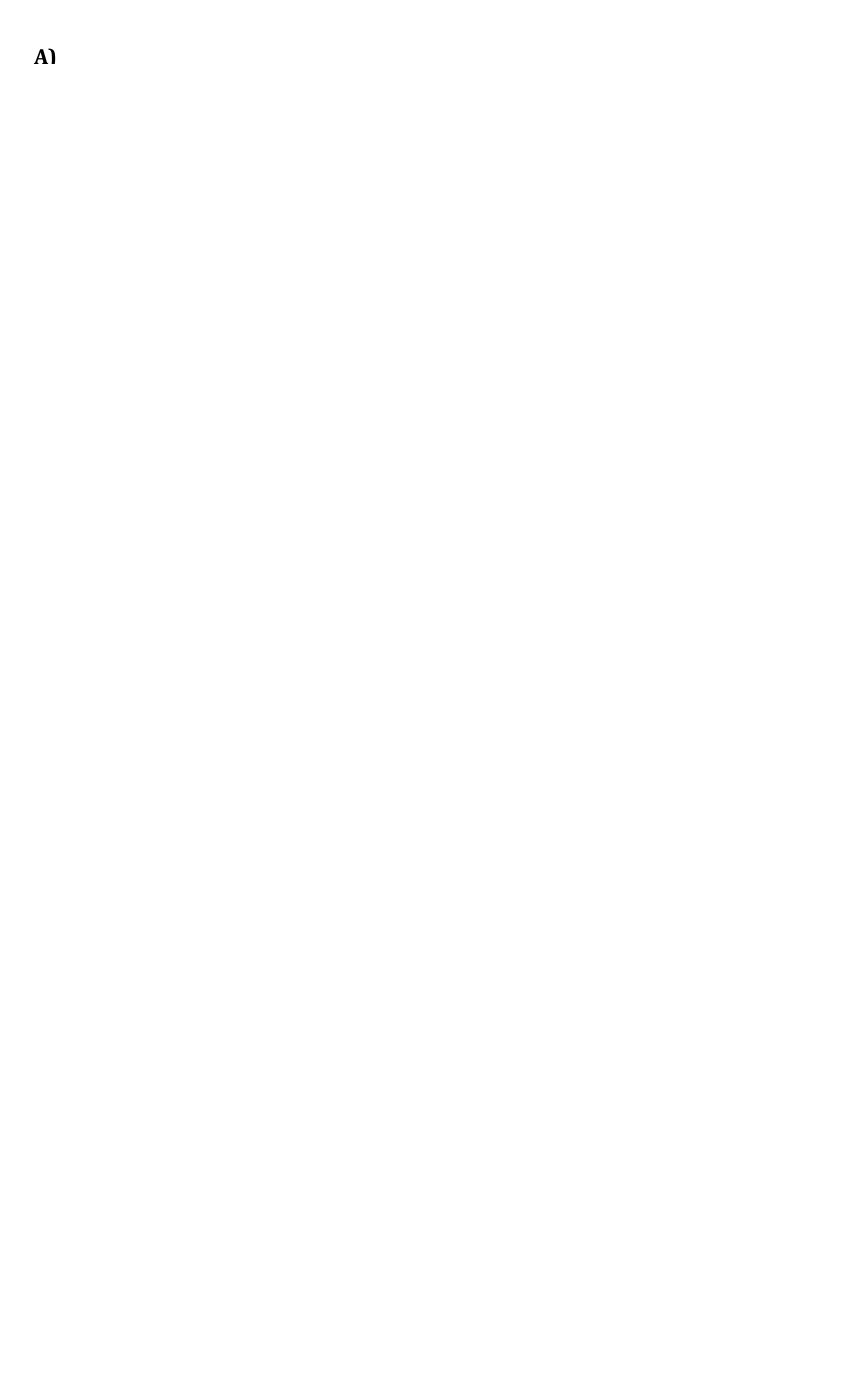
)**

**H)** **
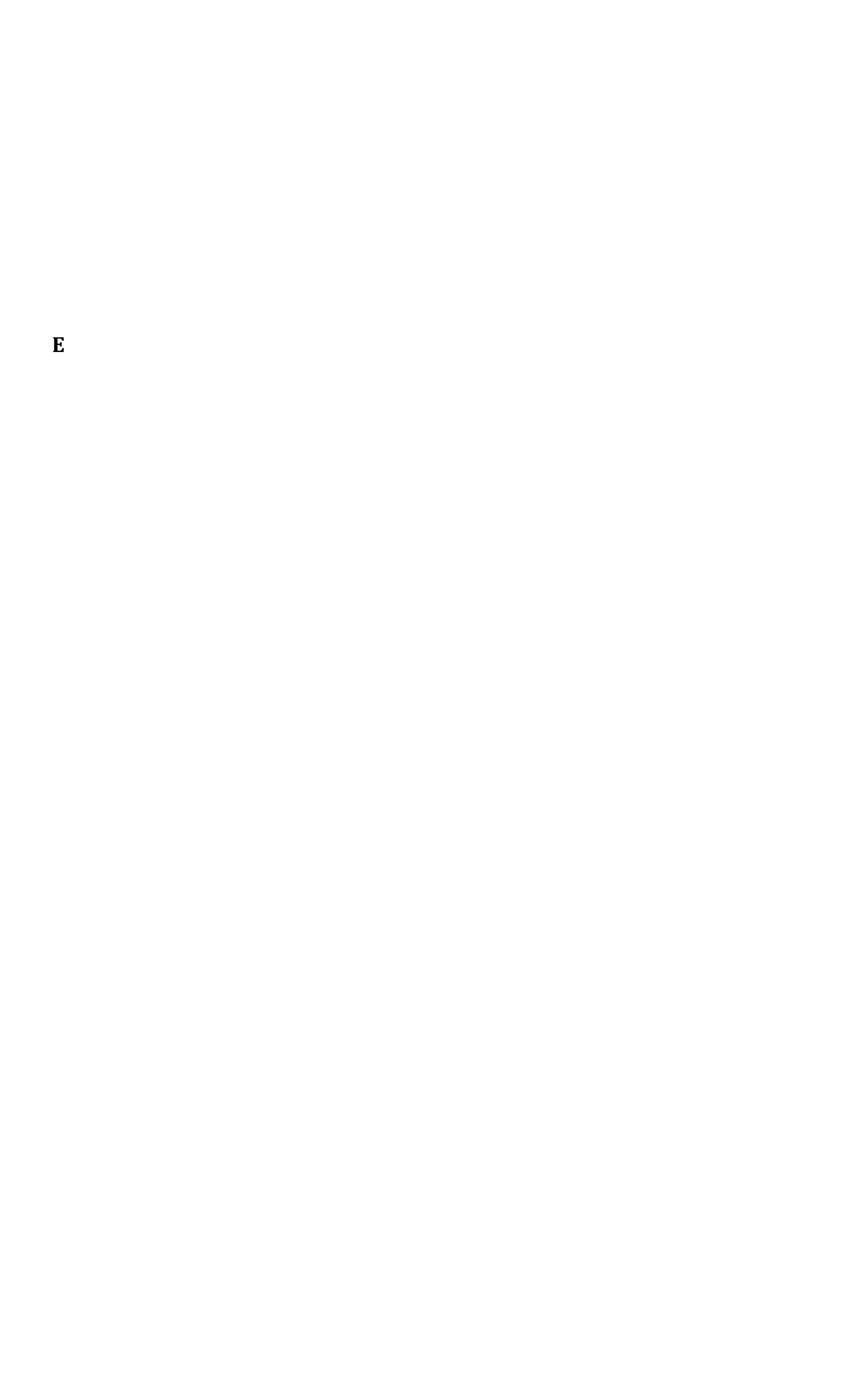

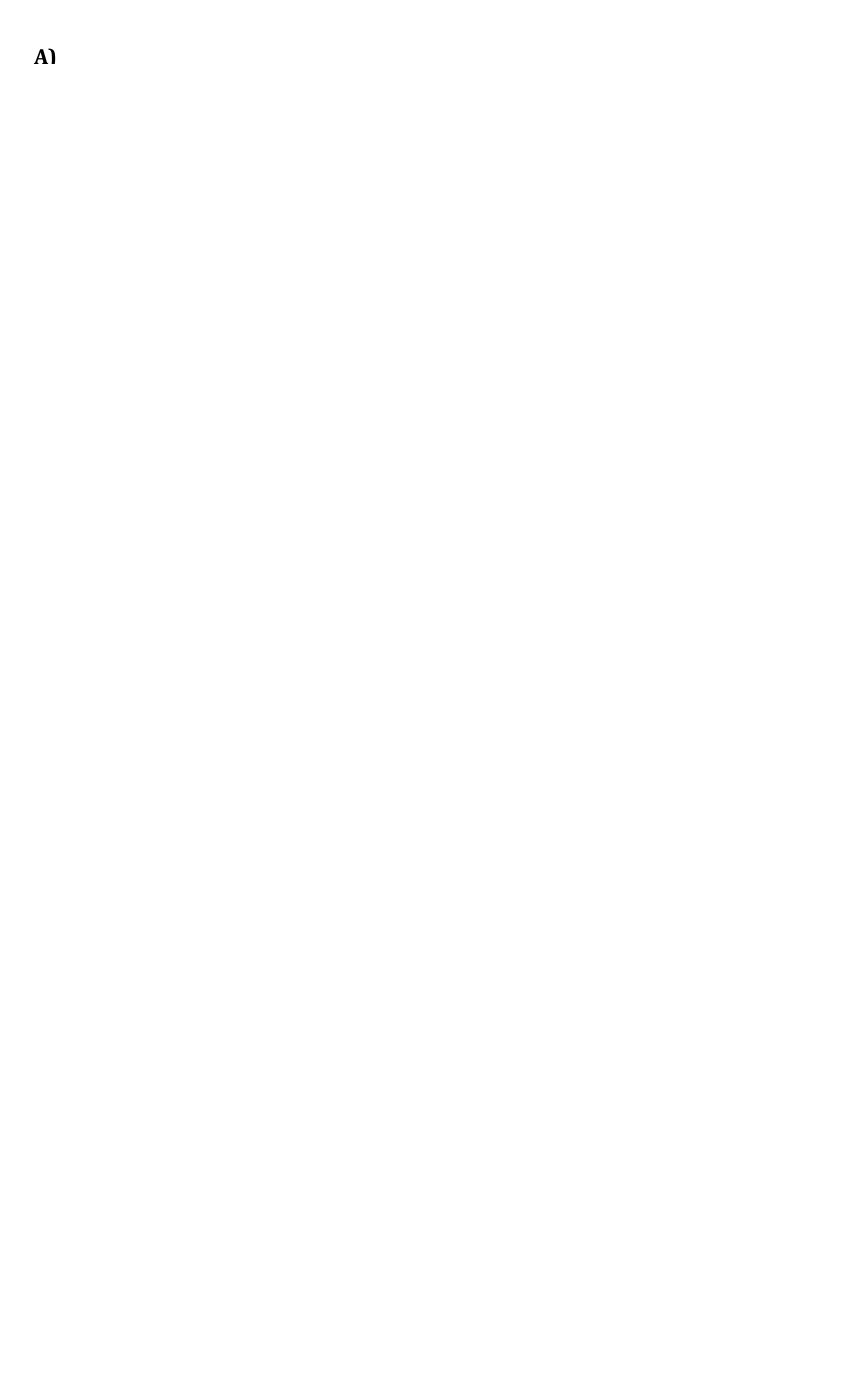
)**

**Figure 2. False discovery rate (FDR) corrected p-values**^a^**.**

**
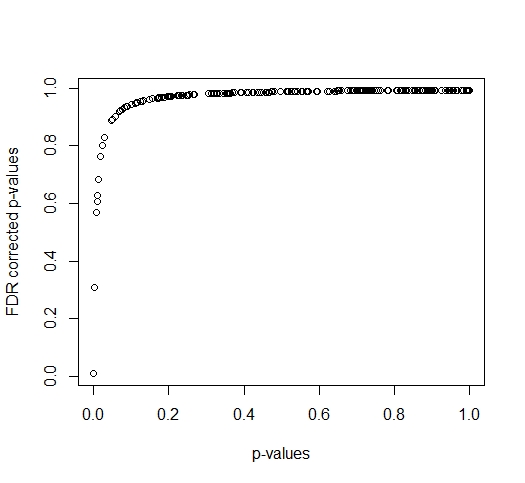
**

^a^ A total of 213 p values have been included, of them 11 were statistically significant and after doing corrections only 1 was significant. All p values from 3 and 5 year lung cancer survival, multiple logistic regression models and toxicity according to presence of genetic polymorphisms and treatment received, have been included.
